# Genome-wide expression profiling in muscle and subcutaneous fat of lambs in response to the intake of concentrate supplemented with vitamin E

**DOI:** 10.1186/s12864-016-3405-8

**Published:** 2017-01-17

**Authors:** Laura González-Calvo, Elda Dervishi, Margalida Joy, Pilar Sarto, Roberto Martin-Hernandez, Magdalena Serrano, Jose M. Ordovás, Jorge H. Calvo

**Affiliations:** 1 Unidad de Tecnología en Producción Animal, CITA, 59059 Zaragoza, Spain; 2 University of Alberta, 116 St and 85 Ave, Edmonton, AB T6G 2R3 Canada; 3 IMDEA-Alimentación, 28049 Madrid, Spain; 4 Departamento de Mejora Genética Animal, INIA, 28040 Madrid, Spain; 5 Jean Mayer-USDA Human Nutrition Research Center on Aging at Tufts University, Boston, MA USA; 6 ARAID, 50004 Zaragoza, Spain

**Keywords:** Vitamin E, Genome-wide expression profiling, Microarray, Sheep, Musclem, Subcutaneous fat, Meat quality

## Abstract

**Background:**

The objective of this study was to acquire a broader, more comprehensive picture of the transcriptional changes in the L. *Thoracis* muscle (LT) and subcutaneous fat (SF) of lambs supplemented with vitamin E. Furthermore, we aimed to identify novel genes involved in the metabolism of vitamin E that might also be involved in meat quality. In the first treatment, seven lambs were fed a basal concentrate from weaning to slaughter (CON). In the second treatment, seven lambs received basal concentrate from weaning to 4.71 ± 2.62 days and thereafter concentrate supplemented with 500 mg dl-α-tocopheryl acetate/kg (VE) during the last 33.28 ± 1.07 days before slaughter.

**Results:**

The addition of vitamin E to the diet increased the α-tocopherol muscle content and drastically diminished the lipid oxidation of meat. Gene expression profiles for treatments VE and CON were clearly separated from each other in the LT and SF. Vitamin E supplementation had a dramatic effect on subcutaneous fat gene expression, showing general up-regulation of significant genes, compared to CON treatment. In LT, vitamin E supplementation caused down-regulation of genes related to intracellular signaling cascade. Functional analysis of SF showed that vitamin E supplementation caused up-regulation of the lipid biosynthesis process, cholesterol, and sterol and steroid biosynthesis, and it down-regulated genes related to the stress response.

**Conclusions:**

Different gene expression patterns were found between the SF and LT, suggesting tissue specific responses to vitamin E supplementation. Our study enabled us to identify novel genes and metabolic pathways related to vitamin E metabolism that might be implicated in meat quality. Further exploration of these genes and vitamin E could lead to a better understanding of how vitamin E affects the oxidative process that occurs in manufactured meat products.

**Electronic supplementary material:**

The online version of this article (doi:10.1186/s12864-016-3405-8) contains supplementary material, which is available to authorized users.

## Background

Vitamin E is a lipid-soluble essential component of human and animal diets due to its powerful antioxidant activity. There are eight different isoforms of vitamin E, and the most active isoform for cell protection appears to be α-tocopherol [1]. Alpha-tocopherol is a cell signaling molecule involved in a broad range of effects on cellular systems [2] and specific regulation of gene expression [2–6]; therefore, α-tocopherol can influence a number of biological functions by regulating cell signaling at both the mRNA and microRNA (miRNA) levels [7]. So far, the impact and beneficial effects of vitamin E in the prevention of chronic diseases have been mainly associated with its antioxidant properties [8].

Vitamin E is extensively used in animal diets as an antioxidant supplement. Feeding strategy is an important tool to manipulate intramuscular α-tocopherol content in animals. An increase in α-tocopherol intake can be achieved through grazing systems [9] or by feeding animals with concentrate supplemented with α-tocopherol, which can be used as an effective method to reduce the oxidative processes reported in meat products [10, 11].

During oxidative processes, the muscle haeminic pigment changes from red oxymyoglobin to brown metmyoglobin, giving the meat an undesirable brownish color [9]. Moreover, lipid oxidation results in the production of free radicals, which are linked to the formation of off-flavors and odors, a reduction in polyunsaturated fatty acids and the production of undesirable compounds, such as potentially toxic peroxides and aldehydes [12]. All of these modifications cause decreases in the freshness and quality of meat and lower consumer acceptance, resulting in considerable economic loss for the meat industry.

So far, efforts to discover the genes and metabolic pathways involved in vitamin E metabolism have mainly focused on rodents. These studies have reported important long-term effects of vitamin E deficiency on liver gene expression, up-regulation of genes involved in cholesterol synthesis and steroidogenesis, lipid uptake, and anti-oxidative mechanisms [3, 13]. In addition, vitamin E in the rat brain affected genes associated with hormones, nerve growth, apoptosis, dopaminergic neurotransmission, and clearance of amyloid-α [14]. Furthermore, genes encoding for muscle structure and extra cellular matrix and those involved in anti-oxidative and anti-inflammatory processes were up-regulated in rat skeletal muscle in response to vitamin E deficiency [15].

Although nutritional science has embraced the tools of genomics, including cDNA arrays in rodent models, few attempts at large-scale or global evaluation of nutritional gene regulation have mainly used rodents as a model. Only a small number of genes are currently known to be related to vitamin E metabolism in muscle or fat. In the last few years, several studies have explored the transcriptomic adaptations of skeletal muscle in response to different nutrition variables in ruminants [16–19]. Data from these studies have allowed scientists to identify the biochemical mechanisms that could be associated with key physiological processes in animals and to define specific markers for evaluating meat quality.

A better understanding of the genes and metabolic pathways associated with vitamin E metabolism is critical for identifying the key physiological processes associated with vitamin E content, oxidative stress and other metabolic pathways associated with meat quality. The main effect of vitamin E on meat is to change the lipid oxidation and thus to increase shelf-life of meat. Because lipid oxidation depends mainly on enzyme activity, post-transcriptional and post-translational changes are processes implicated in the shelf-life of meat. However, the identification of genes related to vitamin E content would be a good starting point for candidate gene selection and single nucleotide polymorphism (SNP) association studies with variations in vitamin E content and therefore implicated in meat quality. Furthermore, non-antioxidant roles of vitamin E, serving as a regulator of gene/protein, have been described. In this sense, transcriptomic analysis of skeletal muscle could identify metabolic pathways modified by vitamin E that could influence meat traits.

Therefore, the main objective of this study was to investigate transcriptional changes in the *L. Thoracis* muscle (LT) and subcutaneous fat (SF) of lambs supplemented with vitamin E using the Affymetrix Ovine Gene 1.1 ST whole-genome array. Furthermore, we aimed to identify novel genes that could play important roles in the metabolism of vitamin E and that might be associated with meat quality traits.

## Results

### Alpha-tocopherol muscle content, intramuscular fat, TBARS and metmyoglobin formation

Significant differences in weaning weight and slaughter age (SA), and average daily gain (ADG), from birth to weaning and from birth to slaughter, were found between treatments (Table 1). Animals from the CON group were younger at slaughter (*P* < 0.05). In addition, these animals had greater ADG from birth to weaning (*P* < 0.01) and from birth to slaughter (*P* < 0.05). During the experimental period, the ADG from weaning to slaughter was not significant between treatments. Average daily gain from birth to slaughter and SA were highly correlated between them (*r* ^2^ = -0.90; *P* < 0.01). The results of α-tocopherol content, intramuscular fat (IMF) content, thiobarbituric acid-reactive substances (TBARS) and metmyoglobin (MMb) formation in the LT muscle for each treatment are shown in Table 1. Our results showed that the content of α-tocopherol in the muscle was significantly higher in lambs that received a basal concentrate supplemented with dl-α-tocopheryl acetate (VE), while lambs fed a basal concentrate (CON) showed higher values of TBARS (mg of malonaldehyde per kg of L. Thoracis) (*P* < 0.05). Furthermore, metmyoglobin formation was significantly lower in VE lambs, compared to the CON group (*P* < 0.05). The K/S_572/525_ ratio decreased when the metmyoglobin content increased. Intramuscular fat (IMF) content was not different between the treatments (*P* > 0.05).

**Table 1:** Effect of the dietary treatment on slaughter age and weight, growth rate, alpha-tocopherol muscle content, intramuscular fat, TBARS and metmyoglobin formation

Treatment^1^				
	CON	VE	SE	*P*-value
Performance				
Weaning Weight (kg)	15.29	11.59	0.5246	**
Slaughter Age (days)	72.28	86.28	3.59	*
Slaughter Weight (kg)	23.20	22.71	0.45	ns
Average Daily Gain (g/day)				
- From birth to weaning	281.43	203.71	11.10	**
- Experimental period^2^	337.86	276.57	22.70	ns
- From birth to slaughter	278.14	230.57	12.25	*
L. Thoracis muscle				
α-Tocopherol, mg/kg muscle	0.13	2.26	0.07	***
IMF (% fresh matter)	2.03	1.68	0.25	ns
TBARS (7 d)^3^	1.62	0.07	0.17	***
K/S_572/525_ (7 d)^4^	0.93	1.06	0.03	*

^1^CON: commercial concentrate without VE supplement; VE: VE concentrate for 33.3 ± 1.07 days before slaughter. Means within a row with different superscripts differ (*P* < 0.05); SE: standard error; ns: not significant; ***P < 0.001; **P < 0.01;*P < 0.05; ^2^Experimental period started at 48 d old. ^3^TBARS: mg of malonaldehyde per kg of L. Thoracis muscle at 7 days of display; ^4^K/S_572/525_: ratio of metmyoglobin formation of L. Thoracis muscle at 7 days of display

### Microarray gene expression results

#### Identification and classification of differentially expressed genes by microarray analysis in LT and SF

**Fig. 1:**
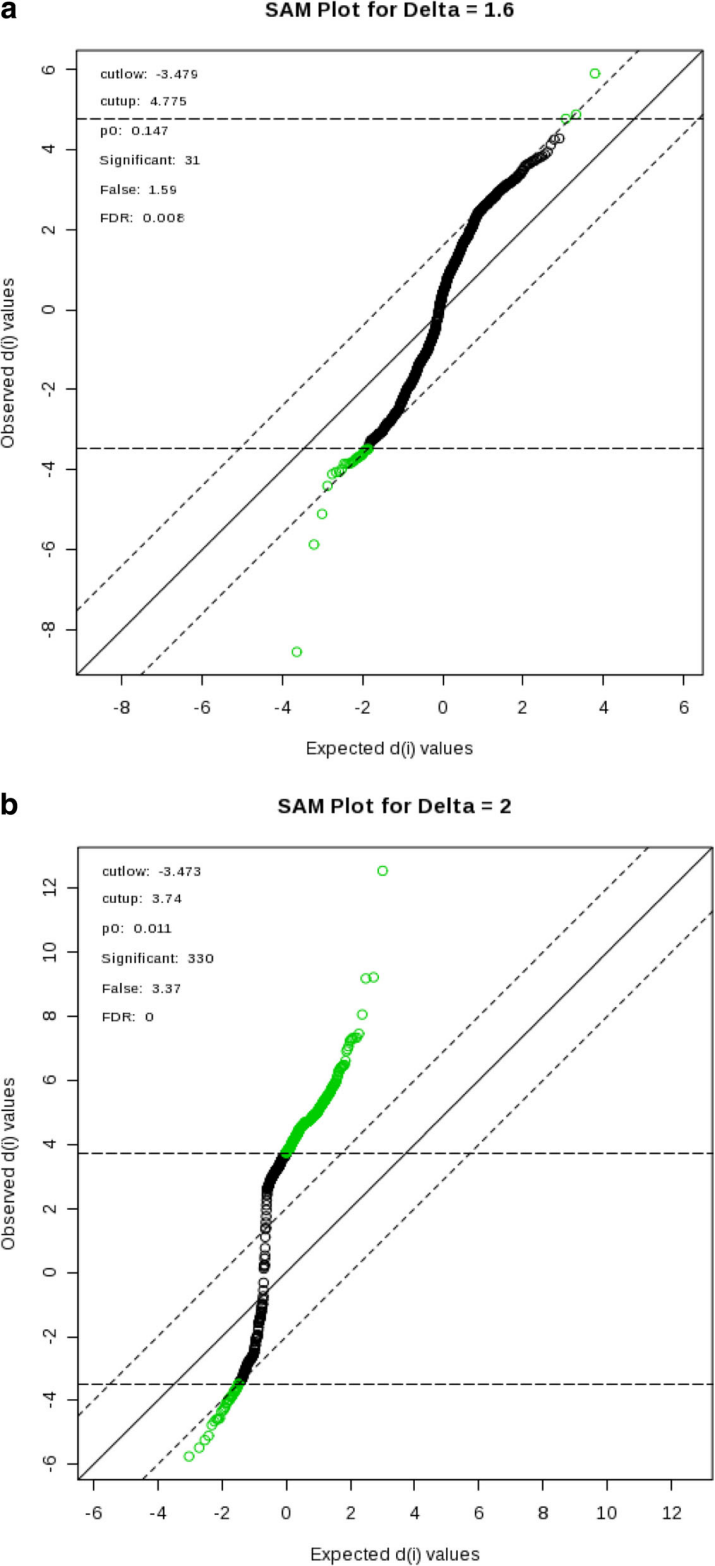
Significant features identified by SAM in **a** VE-CON contrast in LT, and **b** VE-CON contrast in SF. The *green* circles represent features that exceed the specified threshold

Significance analysis of microarray (SAM) was performed to compare the VE treatment with CON. SAM identified a total of 29 genes with a false discovery rate (FDR) = 0.008, 26 down- regulated and 3 up-regulated genes (Table 2). The results of SAM regarding LT muscle are shown in Fig. 1.

**Table 2:** The significant features identified by SAM in VE vs. CON contrast in L. Thoracis muscle

Gene symbol	Gene name	*q* value	FC
*AKR7A2*	aldo-keto reductase family 7, member A2	0	−1.25
*DEF8*	differentially expressed in FDCP 8 homolog	0	−1.20
*ASPN*	asporin	0	1.65
*PPP6R3*	Protein Phosphatase 6, Regulatory Subunit 3	0.00295	−1.14
*SAT1*	spermidine/spermine N1-acetyltransferase 1	0.00295	1.55
*KLHL33*	kelch-like 33	0.00442	−1.30
*ABCC4*	ATP-binding cassette, sub-family C (CFTR/MRP), member 4	0.00925	−1.22
*CISH*	cytokine inducible SH2-containing protein	0.00954	−2.05
*AKAP7*	A kinase (PRKA) anchor protein 7	0.00954	−1.53
*ACACB*	acetyl-Coenzyme A carboxylase beta	0.00954	−1.41
*MAFB*	v-maf musculoaponeurotic fibrosarcoma oncogene homolog B	0.00954	−1.37
*H1F0*	H1 histone family, member 0	0.00954	−1.32
*MYLK2*	myosin light chain kinase 2	0.00954	−1.31
*DUSP26*	dual specificity phosphatase 26 (putative)	0.00954	−1.30
*RSC1A1*	regulatory solute carrier protein, family 1, member 1	0.00954	−1.28
*FHL3*	four and a half LIM domains 3	0.00954	−1.26
*ACAT1*	acetyl-Coenzyme A acetyltransferase 1	0.00954	−1.25
*FAM151A*	family with sequence similarity 151, member A	0.00954	−1.23
*KIAA0430*	KIAA0430	0.00954	−1.22
*ZNF777*	zinc finger protein 777	0.00954	−1.20
*DEPTOR*	DEP Domain Containing MTOR-Interacting Protein	0.00954	−1.19
*LRRTM2*	leucine rich repeat transmembrane neuronal 2	0.00954	1.26
*DDX39B*	DEAD (Asp-Glu-Ala-Asp) Box Polypeptide 39B	0.00974	−1.43
*PGLS*	6-phosphogluconolactonase	0.0097	−1.21
*BTNL9*	butyrophilin-like 9	0.01006	−1.39
*FBXL4*	F-box and leucine-rich repeat protein 4	0.01006	−1.38
*IGF1R*	insulin-like growth factor 1 receptor	0.01006	−1.17
*ST6GALNAC6*	ST6-N-acetylgalactosaminide alpha-2,6-sialyltransferase 6	0.01006	1.14
*ANAPC16*	chromosome 10 open reading frame 104	0.01006	1.25

Regarding subcutaneous fat, when VE treatment was compared with the CON group, SAM identified a total of 330 genes with a FDR < 0.001. Among these genes, 295 were up-regulated, and 35 were down-regulated. The results of the top 50 genes identified with SAM for SF are shown in Table 3. In Additional file 1: Table S1 all of the significant genes in SF are ranked according to their fold change (FC). Notably, *H1F0* gene was found to be significantly down-regulated in VE lambs in both tissues.

**Table 3:** Top 50 genes identify with SAM in VE vs. CON contrast in subcutaneous fat

Gene symbol	Gene Name	*q* value	FC
*DDX47*	DEAD (Asp-Glu-Ala-Asp) box polypeptide 47	0	1.50
*ALG11*	UTP14, U3 small nucleolar ribonucleoprotein, homolog C	0	1.67
*SRPRB*	signal recognition particle receptor, B subunit	0	2.30
*TTC37*	tetratricopeptide repeat domain 37	1.07E-05	1.32
*RRAGA*	Ras-related GTP binding A	1.07E-05	1.40
*LRRC59*	leucine rich repeat containing 59	1.07E-05	1.47
*RASSF8*	Ras association domain family (N-terminal) member 8	1.07E-05	1.55
*ELP3*	elongation protein 3 homolog	1.07E-05	1.56
*SEC23IP*	SEC23 interacting protein	1.07E-05	1.61
*TMEM181*	transmembrane protein 181	1.07E-05	1.65
*CAND1*	cullin-associated and neddylation-dissociated 1	1.53E-05	1.39
*PIGM*	phosphatidylinositol glycan anchor biosynthesis, class M	1.53E-05	1.42
*TMEM203*	transmembrane protein 203	1.53E-05	1.52
*ABCE1*	similar to ATP-binding cassette, sub-family E, member 1	1.53E-05	1.69
*WDR55*	WD repeat domain 55	2.01E-05	1.31
*SLC35A4*	solute carrier family 35, member A4	2.01E-05	1.32
*ARPC5*	actin related protein 2/3 complex, subunit 5, 16 kDa	2.04E-05	1.29
*MINPP1*	multiple inositol polyphosphate histidine phosphatase, 1	2.04E-05	1.38
*RER1*	RER1 retention in endoplasmic reticulum 1 homolog	2.04E-05	1.46
*TIMM22*	translocase of inner mitochondrial membrane 22 homolog	2.04E-05	1.56
*EXTL3*	exostoses (multiple)-like 3	2.04E-05	1.63
*GMPS*	guanine monphosphate synthetase	2.23E-05	1.45
*SLC39A7*	solute carrier family 39 (zinc transporter), member 7	2.23E-05	1.49
*MARS*	methionyl-tRNA synthetase	2.23E-05	1.56
*RAB22A*	RAB22A, member RAS oncogene family	2.47E-05	1.32
*ADSS*	adenylosuccinate synthase	2.47E-05	1.58
*IER3*	immediate early response 3	2.81E-05	−2.06
*RNF121*	ring finger protein 121	2.81E-05	1.34
*DNAJC5*	DnaJ (Hsp40) homolog, subfamily C, member 5	2.81E-05	1.34
*NUDT9*	nudix (nucleoside diphosphate linked moiety X)-type motif 9	2.81E-05	1.36
*WAPAL*	wings apart-like homolog	2.81E-05	1.38
*FBXO28*	F-box protein 28	2.81E-05	1.41
*AP1S1*	adaptor-related protein complex 1, sigma 1 subunit	2.81E-05	1.43
*CNPY4*	canopy 4 homolog	2.81E-05	1.48
*KIAA1274*	Phosphatase Domain Containing, Paladin 1	2.81E-05	1.48
*AKAP10*	A kinase (PRKA) anchor protein 10	2.81E-05	1.48
*HNRNPH2*	ribosomal protein L36a pseudogene 51	2.81E-05	1.52
*MCU*	mitochondrial calcium uniporter	2.81E-05	1.53
*SLC39A3*	solute carrier family 39 (zinc transporter), member 3	2.81E-05	1.57
*EMC8*	ER membrane protein complex subunit 8	2.81E-05	1.59
*PROSC*	proline synthetase co-transcribed homolog	2.81E-05	1.62
*ABCF3*	ATP-binding cassette, sub-family F (GCN20), member 3	2.81E-05	1.65
*C5H22orf28*	chromosome 5 open reading frame, human C22orf28	2.81E-05	1.67
*PINX1*	PIN2-interacting protein 1	2.81E-05	1.69
*ADPGK*	ADP-dependent glucokinase	2.81E-05	1.75
*CERS6*	ceramide synthase 6	2.81E-05	1.76
*DOLPP1*	dolichyl pyrophosphate phosphatase 1	2.81E-05	1.79
*DOLK*	dolichol kinase	2.81E-05	1.86
*RPS4Y1*	ribosomal protein S4, Y-linked 1	2.81E-05	1.90
*TIMM8A*	translocase of inner mitochondrial membrane 8 homolog A	2.81E-05	2.27

#### Treatment-dependent multivariate analysis results of gene expression

**Fig. 2:**
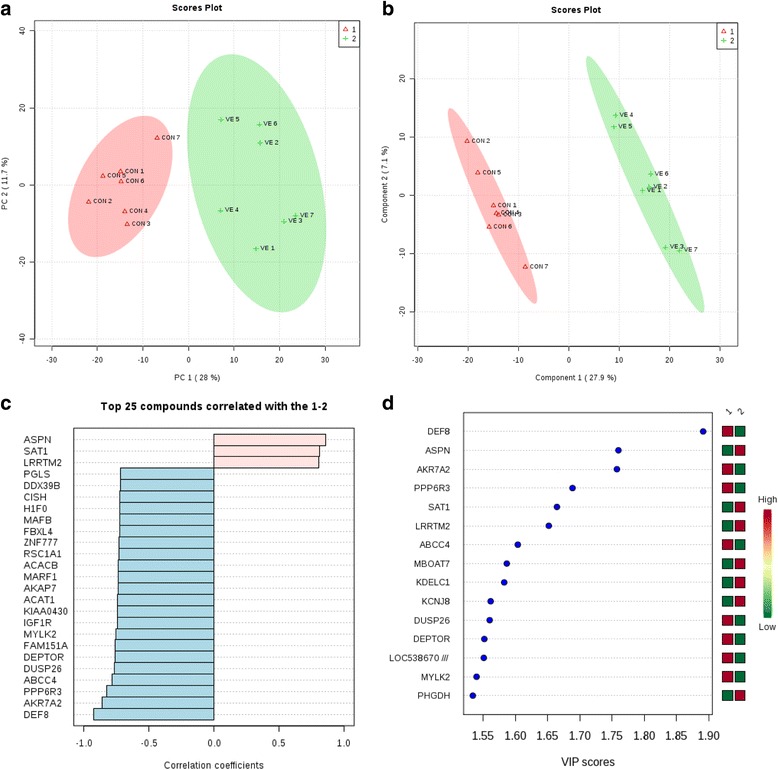
Multivariate analysis based on gene expression profile data in LT muscle. **a** Principal component analysis (PCA) score plots distinguishing between the LT muscle of lambs fed concentrate (triangle), and supplemented with vitamin E in the treatment, (+). **b** Partial least squares-discriminant analysis (PLC-DA) based on gene expression profile data. **c** Important features identified by PLS-DA. The top 15 genes are ranked by VIP scores. The colored boxes on the right indicate the relative expression of the corresponding gene in each group under study. **d** A bar graph showing the top 25 genes correlating with diet (CON, VE). Variables with the same distance from 0 with similar positions are positively correlated. Those with the opposite direction are negatively correlated. The *light blue* bars indicate genes showing a negative correlation, and the *light pink* bars indicate those with a positive correlation with the given pattern of change of treatment

In the LT muscle, principal component analysis (PCA) of the complete set of 32 genes identified by SAM showed that the first 2 PCs covered 39.7% of the observed variance in the sample set (Fig. 2a). The PCA score plot revealed differences corresponding to lambs fed with the two different treatments. The ellipse corresponding to CON was clearly separated from the VE treatment. Partial least squares-discriminate analysis (PLS-DA) showed a clear separation of the two groups (Fig. 2b). In addition, PLS-DA allowed for the identification of the genes that were most important for the separation observed in the score plots. *DEF8* gene showed the highest score, followed by *ASPN* and *AKR7A2* (Fig. 2c). Moreover, we investigated trends or patterns in gene expression changes (Fig. 2d). For example, *ABCC4*, *DEPTOR*, *IGFR1*, *MYLK2* and *ACAT1* were positively correlated with each other in the two treatments, showing a down- and up-regulation in the VE and CON treatments, respectively. In contrast, they were negatively correlated with *SAT1*, *ASPN*, *or LRRTM2*. These genes, which are either positively or negatively correlated with each other, appeared to play an important role in light of the PCA and PLS-DA cluster analysis.

**Fig. 3:**
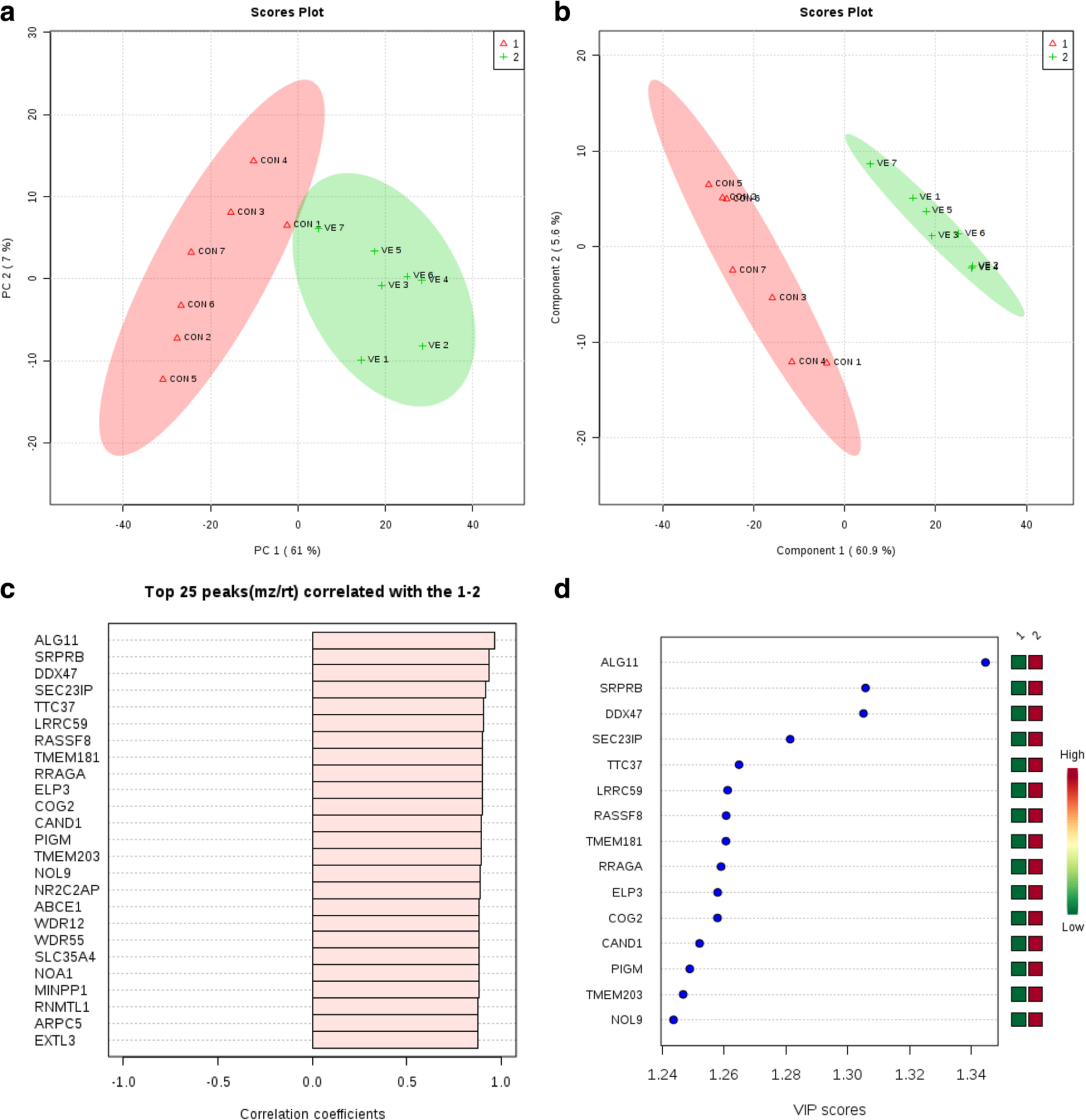
Multivariate analysis based on gene expression profile data in subcutaneous fat. **a** Principal component analysis (PCA) score plots distinguishing between the subcutaneous fat of lambs fed concentrate (triangle), and supplemented with vitamin E in the treatment (+). **b** Partial least squares-discriminant analysis (PLC-DA) based on gene expression profile data. **c** Important features identified by PLS-DA. The top 15 genes are ranked by VIP scores. The colored boxes on the right indicate the relative expression of the corresponding gene in each group under study. **d** A bar graph showing the top 25 genes correlating with treatment (CON, VE). Variables with the same distance from 0 with similar positions are positively correlated. Those with the opposite direction are negatively correlated The *light pink* bars indicate genes with a positive correlation with the given pattern of change of treatment

Multivariate analysis results from subcutaneous fat were used to cluster the samples based on gene expression profiles of animals fed two different diets: CON and VE. The PCA of the complete set of 330 genes identified by SAM showed that the first 2 PCs covered 68% of the observed variance of the sample set (Fig. 3a). The figure shows the score plot of the two first principal components extracted in this study. PLS-DA showed that the VE and CON groups were clearly separate from each other. (Fig. 3b). In addition, PLS-DA allowed for the identification of the most important genes contributing to the separation observed in the score plots, and *ALG11* had the highest score, followed by *SRPBR* and *PPX47*.

Moreover, we investigated patterns in gene expression changes in subcutaneous fat (Fig. 3d). For example, the gene expressions of *ALG11*, *SRPBR*, and *PPX47* were positively correlated with each other, being up-regulated in VE treatment.

#### Hierarchical clustering analysis (HCA)

**Fig. 4:**
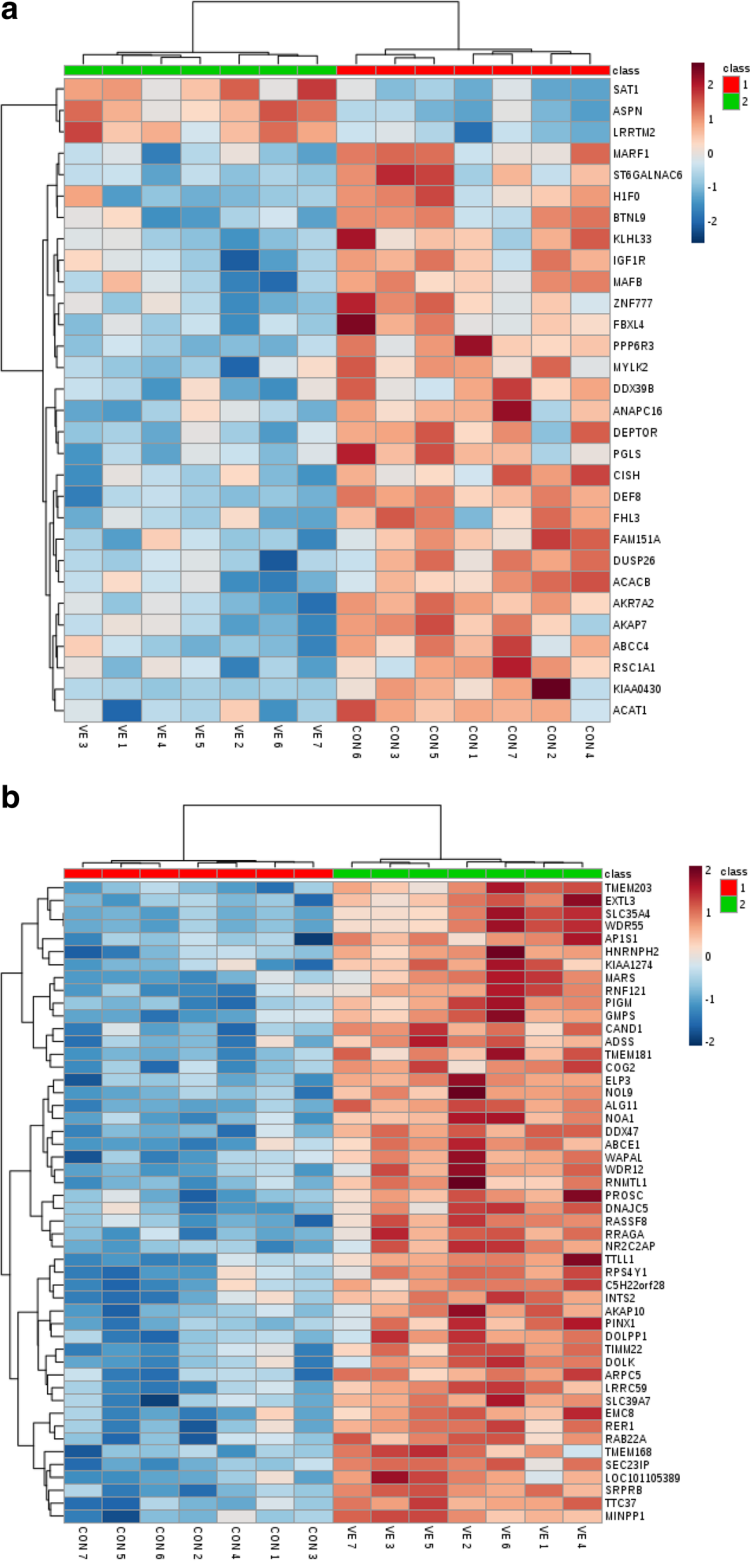
Hierarchical clustering analysis of gene expression in **a**) the L. Thoracis muscle, **b**) subcutaneous fat tissue of lambs receiving different treatments (CON and VE), using the most significant genes of each contrast. Cells are colored based on the signal intensity measured. *Dark brown* represents high gene expression levels, *blue* indicates low signal intensity, and *gray* indicates the intermediate level (see color scale above the heatmap)

HCA was performed using the significant genes obtained by SAM for both contrasts. The results of HCA for LT muscle are presented in Fig. 4a. The expression profile of these genes was able to cluster and to classify correctly the samples within their corresponding groups. The heatmap shows the presence of 2 different clusters containing different genes. The responses of each variable to the two different treatments are indicated with changes in the color intensity on the heatmap. The VE and CON groups showed very different gene expression patterns. For instance, *LRRTM2*, *ASPN* and *SAT1* were up-regulated in the VE group. Furthermore, a second cluster including the remainder of the genes was found to be down-regulated in the VE group. These two clusters distinguished the VE group from the CON group. These genes are involved in different metabolic processes.

Regarding subcutaneous fat, the results of clustering analysis of top 50 genes are presented in Fig 4b. Analyzing the heatmap, it can be observed that the CON group showed very different gene expression patterns from the VE group. There is a general down-regulation of gene expression in the CON group and an up-regulation of gene expression in the VE group. The results of clustering analysis of the total 330 genes in subcutaneous fat were similar to the 50 genes analysis results, but one VE animal was clustered in the CON group (Additional file 2: Figure S1). More specifically, 34 genes were down- regulated in the VE group (*SOD3*, *CLEC3B*, *METTL7A*, *IER3*, *PLK2*, *CLEC1A*, *HSPA1A*, *TNFSF10*, *MERTK*, *MLLT3*, *TPPP2*, *HMGB2*, *PECAM1*, *IFITM3*, *H1F0*, *HMG20B*, *GALK1*, *NFIL3*, *CDH5*, *ERG*, *CEBPA*, *ANKRD44*, *HCST*, *UACA*, *IFITM1*, *RPL10*, *PID1*, *LUC7L3*, *H3F3A*, *AMOTL2*, *PSIP1*, *RPL17*, *TRA2A*, and *RPS259*).

### Functional clustering annotation

In the LT muscle, the results of Database for Annotation, Visualization and Integrated Discovery (DAVID) functional annotation clustering (FAC), including the 29 significant genes in the VE-CON contrast, showed that 4 genes from the “intracellular signaling cascade” (*IGF1R*, *DEF8*, *AKAP7* and *CISH*) had the most enrichment and were all down-regulated in the VE treatment (Additional file 3: Table S2). However, the confident enrichment scores were less than 1.3 in both cases.

In subcutaneous fat, the results of DAVID FAC of 330 significant genes in the VE-CON contrast, revealed that the most significantly enriched cluster was “lipid biosynthesis process”, (enrichment score of 5.38) with a total of 20 genes involved (*PGAP3*, *EBP*, *CRLS1*, *MVD*, *CYP51A1*, *GNE*, *HMGCS1*, *DPAGT1*, *LSS*, *SIGMAR1*, *LPCAT3*, *FDFT1*, *DOLK*, *PIGM*, *SQLE*, *DHCR7*, *AGPAT9*, *LTA4H*, *PCYT2* and *HSD17B7*), all of them up-regulated in the VE group compared to the CON group. Many of these genes play roles in the cholesterol, sterol and steroid biosynthesis. In Table 4, the DAVID FAC results of the 330 genes in the VE-CON contrast are shown.

**Table 4:** DAVID Functional Annotation Clustering of SAM genes in VE vs. CON subcutaneous fat

Annotation Cluster 1					
Enrichment Score: 5.82					
Category	Term	Count	%	*P* Value	Genes
GOTERM_BP_FAT	GO:0016126 ~ sterol biosynthetic process	9	3.26	6.03E-08	*EBP*, *MVD*, *CYP51A1*, *SQLE*, *DHCR7*, *HMGCS1*, *LSS*, *SIGMAR1*, *FDFT1*
GOTERM_BP_FAT	GO:0016125 ~ sterol metabolic process	12	4.35	6.02E-07	*SREBF1*, *EBP*, *LDLR*, *MVD*, *CYP51A1*, *SQLE*, *DHCR7*, *INSIG1*, *HMGCS1*, *LSS*, *SIGMAR1*, *FDFT1*
GOTERM_BP_FAT	GO:0008610 ~ lipid biosynthetic process	20	7.25	1.04E-06	*PGAP3*, *EBP*, *CRLS1*, *MVD*, *CYP51A1*, *GNE*, *HMGCS1*, *DPAGT1*, *LSS*, *SIGMAR1*, *LPCAT3*, *FDFT1*, *DOLK*, *PIGM*, *SQLE*, *DHCR7*, *AGPAT9*, *LTA4H*, *PCYT2*, *HSD17B7*
GOTERM_BP_FAT	GO:0008203 ~ cholesterol metabolic process	11	3.99	2.01E-06	*SREBF1*, *EBP*, *LDLR*, *MVD*, *CYP51A1*, *SQLE*, *DHCR7*, *INSIG1*, *HMGCS1*, *LSS*, *FDFT1*
GOTERM_BP_FAT	GO:0006695 ~ cholesterol biosynthetic process	7	2.54	2.73E-06	*EBP*, *MVD*, *CYP51A1*, *DHCR7*, *HMGCS1*, *LSS*, *FDFT1*
GOTERM_BP_FAT	GO:0006694 ~ steroid biosynthetic process	10	3.62	8.22E-06	*EBP*, *MVD*, *CYP51A1*, *SQLE*, *DHCR7*, *HMGCS1*, *LSS*, *SIGMAR1*, *HSD17B7*, *FDFT1*
GOTERM_BP_FAT	GO:0008202 ~ steroid metabolic process	13	4.71	9.78E-05	*SREBF1*, *EBP*, *MVD*, *LDLR*, *CYP51A1*, *HMGCS1*, *LSS*, *SIGMAR1*, *FDFT1*, *SQLE*, *DHCR7*, *INSIG1*, *HSD17B7*
Annotation Cluster 2					
Enrichment Score: 3.59					
GOTERM_BP_FAT	GO:0007005 ~ mitochondrion organization	13	4.71	2.11E-06	*NDUFAF4*, *CAV2*, *DNM1L*, *MTX2*, *TOMM40*, *TIMM44*, *TIMM8A*, *TRNT1*, *GFM2*, *MRPL12*, *FXC1*, *PTCD2*, *DNAJA3*
GOTERM_BP_FAT	GO:0015031 ~ protein transport	29	10.51	3.19E-05	*SNX19*, *MTX2*, *SNX4*, *RAB1A*, *COPB2*, *AP1S1*, *FXC1*, *VPS4A*, *NUP35*, *SEC23IP*, *ZW10*, *RAB2B*, *SRP54*, *VPS45*, *TOMM40*, *NUP85*, *AP4S1*, *TIMM44*, *TIMM8A*, *TIMM22*, *TRNT1*, *YWHAG*, *PLEKHA8*, *RAB22A*, *KPNA6*, *NOP58*, *NUP43*, *KPNA1*, *SNX11*
GOTERM_BP_FAT	GO:0045184 ~ establishment of protein localization	29	10.51	3.75E-05	*SNX19*, *MTX2*, *SNX4*, *RAB1A*, *COPB2*, *AP1S1*, *FXC1*, *VPS4A*, *NUP35*, *SEC23IP*, *ZW10*, *RAB2B*, *SRP54*, *VPS45*, *TOMM40*, *NUP85*, *AP4S1*, *TIMM44*, *TIMM8A*, *TIMM22*, *TRNT1*, *YWHAG*, *PLEKHA8*, *RAB22A*, *KPNA6*, *NOP58*, *NUP43*, *KPNA1*, *SNX11*
GOTERM_BP_FAT	GO:0008104 ~ protein localization	31	11.23	6.84E-05	*SNX19*, *MTX2*, *SNX4*, *AKAP10*, *RAB1A*, *COPB2*, *AP1S1*, *FXC1*, *PIKFYVE*, *VPS4A*, *NUP35*, *SEC23IP*, *ZW10*, *RAB2B*, *SRP54*, *VPS45*, *TOMM40*, *NUP85*, *AP4S1*, *TIMM44*, *TIMM8A*, *TIMM22*, *TRNT1*, *YWHAG*, *PLEKHA8*, *RAB22A*, *KPNA6*, *NOP58*, *NUP43*, *KPNA1*, *SNX11*
GOTERM_BP_FAT	GO:0033365 ~ protein localization in organelle	11	3.99	1.17E-04	*TRNT1*, *SRP54*, *MTX2*, *FXC1*, *PIKFYVE*, *KPNA6*, *TOMM40*, *NOP58*, *TIMM44*, *KPNA1*, *TIMM8A*
GOTERM_BP_FAT	GO:0006626 ~ protein targeting to mitochondrion	6	2.17	1.89E-04	*TRNT1*, *MTX2*, *FXC1*, *TOMM40*, *TIMM44*, *TIMM8A*
GOTERM_BP_FAT	GO:0070585 ~ protein localization in mitochondrion	6	2.17	1.89E-04	*TRNT1*, *MTX2*, *FXC1*, *TOMM40*, *TIMM44*, *TIMM8A*
GOTERM_BP_FAT	GO:0034613 ~ cellular protein localization	18	6.52	3.25E-04	*SRP54*, *VPS45*, *MTX2*, *TOMM40*, *AP4S1*, *TIMM44*, *TIMM8A*, *TRNT1*, *COPB2*, *YWHAG*, *AP1S1*, *FXC1*, *PIKFYVE*, *KPNA6*, *NOP58*, *SEC23IP*, *KPNA1*, *ZW10*
GOTERM_BP_FAT	GO:0070727 ~ cellular macromolecule localization	18	6.52	3.51E-04	*SRP54*, *VPS45*, *MTX2*, *TOMM40*, *AP4S1*, *TIMM44*, *TIMM8A*, *TRNT1*, *COPB2*, *YWHAG*, *AP1S1*, *FXC1*, *PIKFYVE*, *KPNA6*, *NOP58*, *SEC23IP*, *KPNA1*, *ZW10*
Annotation Cluster 3					
Enrichment Score: 3.00					
GOTERM_BP_FAT	GO:0008299 ~ isoprenoid biosynthetic process	5	1.81	2.51E-04	*MVD*, *HMGCS1*, *DPAGT1*, *FDFT1*, *DOLK*
GOTERM_BP_FAT	GO:0006720 ~ isoprenoid metabolic process	6	2.17	6.48E-04	*RDH11*, *MVD*, *HMGCS1*, *DPAGT1*, *FDFT1*, *DOLK*
GOTERM_BP_FAT	GO:0016090 ~ prenol metabolic process	3	1.09	2.46E-3	*DPAGT1*, *FDFT1*, *DOLK*
GOTERM_BP_FAT	GO:0016093 ~ polyprenol metabolic process	3	1.09	2.46E-3	*DPAGT1*, *FDFT1*, *DOLK*
Annotation Cluster 4					
Enrichment Score: 1.72					
GOTERM_BP_FAT	GO:0034660 ~ ncRNA metabolic process	13	4.71	3.28E-04	*GARS*, *EPRS*, *IARS*, *TRNT1*, *WDR55*, *NOLC1*, *METTL1*, *ADAT2*, *NOP58*, *ALG11*, *NSUN2*, *FTSJ2*, *MARS*
GOTERM_BP_FAT	GO:0034470 ~ ncRNA processing	9	3.26	1.03E-2	*TRNT1*, *WDR55*, *NOLC1*, *METTL1*, *ADAT2*, *NOP58*, *ALG11*, *FTSJ2*, *NSUN2*
GOTERM_BP_FAT	GO:0042254 ~ ribosome biogenesis	6	2.17	4.59E-2	*WDR55*, *TSR1*, *NOLC1*, *NOP58*, *ALG11*, *FTSJ2*
Annotation Cluster 5					
Enrichment Score: 1.68					
GOTERM_BP_FAT	GO:0006399 ~ tRNA metabolic process	8	2.90	2.83E-3	*TRNT1*, *IARS*, *METTL1*, *ADAT2*, *GARS*, *EPRS*, *NSUN2*, *MARS*
GOTERM_BP_FAT	GO:0043039 ~ tRNA aminoacylation	4	1.45	3.69E-2	*IARS*, *GARS*, *EPRS*, *MARS*
GOTERM_BP_FAT	GO:0043038 ~ amino acid activation	4	1.45	3.69E-2	*IARS*, *GARS*, *EPRS*, *MARS*
GOTERM_BP_FAT	GO:0006418 ~ tRNA aminoacylation for protein translation	4	1.45	3.69E-2	*IARS*, *GARS*, *EPRS*, *MARS*
Annotation Cluster 6					
Enrichment Score: 1.43					
GOTERM_BP_FAT	GO:0016192 ~ vesicle-mediated transport	20	7.25	2.23E-3	*CAV2*, *RAB2B*, *LDLR*, *CNIH*, *VPS45*, *GARS*, *RER1*, *SNX4*, *AP4S1*, *RAB1A*, *COPB2*, *AP1S1*, *TFRC*, *CDC42SE2*, *ACTR1A*, *PIKFYVE*, *RAB22A*, *VPS4A*, *TRAPPC3*, *ZW10*
GOTERM_BP_FAT	GO:0016044 ~ membrane organization	12	4.35	4.24E-2	*CAV2*, *COPB2*, *AP1S1*, *DNM1L*, *LDLR*, *TFRC*, *CDC42SE2*, *FXC1*, *RAB22A*, *PIKFYVE*, *SNX4*, *TIMM8A*

### Validation of microarrays results using qPCR

Thirteen genes were selected to validate the microarray results by quantitative real-time PCR (qPCR). The gene set included 4 genes in muscle and 9 genes in subcutaneous fat. In LT muscle, the genes were selected because they were significant differentially expressed between the VE and CON treatments (*CISH*, *ABCC4*, *ACAT1* and *IGF1R*). In SF, the significant genes in the VE-CON contrast (*LDLR*, *SOD3*, *SQLE*, *SREBF1*, *MTTL1*, *HAX1*, *HMGB2*, *HSPB8* and *IER3*) were selected. Although the magnitude of FC obtained by microarray and qPCR was slightly different in some instances, the qPCR results demonstrated a similar trend compared with the microarray results of these 13 genes, demonstrating the reliability of microarray analysis (Pearson’s correlation coefficient 0.99, *P* = 0.008 for LT muscle and 0.99, *P* < 0.001 for SF) (Table 5).

**Table 5:** Real-time PCR confirmation of the microarray results

	Microarray	qPCR
Gene	VE vs CON	VE vs CON
*L.Thoracis*		
*CISH*	−2.05***	−3.07**
*ABCC4*	−1.22***	−1.07
*ACAT1*	−1.25***	−1.42
*IGF1R*	−1.17**	−1.03
*Subcutaneous fat*		
*LDLR*	2.66****	2.25
*METLL1*	1.53****	1.28
*SOD3*	−2.78****	−3.51**
*SQLE*	2.70****	2.24
*SREBF1*	1.76****	1.73
*HAX1*	1.44****	1.21
*HMGB2*	−1.53****	−2.24
*HSPB8*	1.87****	1.86
*IER3*	−2.06****	−2.54*

Gene expression changes in *L. Thoracis* muscle (*CISH*, *ABCC4*, *ACAT1* and *IGF1R*) and subcutaneous fat (*LDLR*, *SOD3*, *SQLE*, *SREBF1*, *HAX1*, *METLL1*, *HMGB2*, *HSPB8* and *IER3*) by the fold change (FC) obtained with microarray and qPCR data and their significance (*P* < 0.1*; *P* < 0.05**; *P* < 0.01***; *P* < 0.001****)

## Discussion

The aim of the present study was to assess the effects of adding VE to the fattening concentrate, fed between weaning and slaughter, on the transcriptional changes in the LT and SF. The experimental period ended when the lambs reached the target slaughter LW (22–24 kg), according to the specifications of Ternasco de Aragón Protected Geographical Indication (Regulation (EC) No. 1107/96). The light lamb production is based on two periods: suckling and fattening. The suckling period is usually limited by the time, usually lasting approximately 45 d (in the present study, it was 48 d), while the fattening period is limited by the weight (22–24 kg LW). Thus, lambs with high ADG during lactation period spent fewer days in the fattening period than those with low ADG. To be able to evaluate the potential effects of the inclusion of VE on the transcriptome of the *L. Thoracis* muscle and subcutaneous fat, we planned to feed at least 30 days of VE concentrate. According to Ripoll et al. [11], supplementation with VE for 30 days was found to increase noticeably concentration of the α-tocopherol content in muscle. In the present study, the ADG during the experimental period was not different, however during the suckling period it was lower in VE treatment (*p* < 0.01; Table 1). This implies that the lambs of the VE treatment during the suckling period presented lower growth compared to the CON group and needed more days to reach the target weight. Moreover, there was a strong correlation between ADG from birth to slaughter and SA (*r* ^2^ =−0.9). Therefore, to overcome this unbalance between treatments ADG values were included as covariates in the statistical model used to validate gene expression differences by qPCR, thus avoiding estimation biases. We analyzed LT muscle and SF because meat cuts for human consumption include both intramuscular and subcutaneous fat [20], constituting the total amount of meat fat purchased at retail. The results related to the content of α-tocopherol in the muscle and TBARS confirm what was planned in the methodology. Vitamin E has been successfully used to increase the muscle α-tocopherol and to reduce lipid oxidation in beef and chicken meat [21]. In this study, as expected, there were significant differences between both diets in the α-tocopherol content, lipid oxidation and MMb formation in the LT muscle, as previously found by Ripoll et al. [11]. Moreover, TBARS values were lower in VE lambs which showed that the lipid oxidation process was slower in animals fed this type of diet.

In the present study, we used microarray technology to study changes in gene expression profiles in LT muscle and SF in response to vitamin E supplementation in lambs. Our results, showed that vitamin E supplementation caused different responses in gene expression in LT muscle and SF, suggesting a specific response of tissue to vitamin E supplementation. In our study, we did not measure the concentration of VE in the SF, however the gene expression results might be related to the greater α-tocopherol accumulation in adipose tissues than in skeletal muscles [22, 23].

It has been reported that α-tocopherol can influence a number of biological functions by regulating cell signaling at both the mRNA and miRNA levels [7]. Indeed, our results showed that vitamin E supplementation affected the expression of 29 genes in the LT muscle. The results of functional analysis showed that genes related to the intracellular signaling cascade (*CISH*, *IGF1R*, *DEF8*, and *AKAP7*) and metabolic processes (*ZNF79*, *MAFB*, *MYLK2*, *ACACB*, *ACAT1*, *CISH*, *IGF1R*, *PGLS*, *DUSP26*, *AKR7A2*, *FBXL4*, *AKAP7*, and *RSC1A1*) were down-regulated. The enrichment scores were less than 1.3, likely because the number of significant genes in this contrast was low. The most down-regulated gene by vitamin E in LT was *CISH* (Cytokine Inducible SH2 containing protein). Chen et al. [24] reported that CISH activates protein kinase C (PKC) activity by G-protein coupled receptor protein, which is important for the activation of both the activator protein 1 (AP-1) and nuclear factor- κB (NF-κB) pathways. In a previous study Boscoboinik et al. [25], demonstrated that PKC is inhibited by α-tocopherol. NF-κB proteins are a family of transcription factors and are of central importance in inflammation, immunity and apoptosis [26]. Evidence suggests a role for reactive oxidative intermediates (ROIs) as a common and critical intermediate for various NF-κB-activating signals, based on inhibition of NF-κB activation by a variety of antioxidants [27, 28]. There is a bidirectional relationship between cytokines and oxidative stress. Exposure of myotubes to reactive oxygen species (ROS)-producing agents resulted in an increase in interleukin 6 (IL-6) release through the activation of the redox-sensitive transcription factor, NF-κB [28]. In this sense, *IL*-*6* induces marked increases in expression of *CISH*, *SOCS*-*1*, *SOCS*-*2*, and *SOCS*-*3* in tissues, which in turn result in inhibition of the signaling of wide range of cytokines [29]. Thus, *CISH* expression could be related to oxidative status in the muscle. In this sense, low levels of ROS and ROIs in muscle caused by α-tocopherol could be associated with low expression of *CISH*. In this sense, lipid oxidation in the LT muscle and metmyoglobin formation were lower when lambs were supplemented with VE. In the same manner, *RSC1A*, which inhibits a dynamin and PKC-dependent exocytotic pathway of *SLC5A1* gene, was down-regulated in the VE group. Interestingly enough *CISH* and *MYLK*, also down-regulated in the VE treatment, are involved in inflammation mediated by chemokine and cytokines signaling pathway, which could suggest another possible role for VE that of decreased inflammation.

In our study, alterations of the Ras homolog gene family, member A (RhoA) and actin cytoskeleton signaling were identified (*IGF1R* and *MYLK2* genes). Both *IGF1R* and *MYLK2* were down-regulated in VE treatment. Insulin-like growth hormone 1 (IGF-1) is a protein structurally similar to insulin, and it regulates tissue growth and development in several vertebrates [30]. As a main receptor of IGFs, IGF1R mediates the transduction of metabolic signals of cell proliferation, bone growth, and protein synthesis in the GH/IGF pathway [31]. In our study, *IGF1R* was down-regulated in VE treatment, and because of *IGF1R* polymorphisms have been associated to growth traits [32–36], we thought that this effect could be due to the higher ADG in CON animals. However, ADG values were included as a covariate in the statistical model used to validate expression differences by qPCR, thus avoiding estimation biases related to ADG. Therefore these results suggest that supplementation with VE causes down-regulation of *IGF1R* in LT muscle. Our findings are in agreement with Araujo et al. [37], who showed that VE supplementation reduces *IGF1R* expression by 17% in hyperthyroid of Wistar rats. In addition, Chuang et al. [38] also found that VE alone significantly and dose-dependently reduced the cell surface expression of *IGFIR* in HL-60 cells. Moreover, Holzenberger et al. [39] reported that *Igf1r* ^+/−^mice display greater resistance to oxidative stress.

In addition, vitamin E down-regulated two genes related to lipid metabolisms (*ACAT1* and *ACACB*) in the LT muscle. *ACAT1* (acetyl-coenzyme A acetyltransferase 1) encodes a mitochondrial localized enzyme that catalyzes the reversible formation of acetoacetyl- CoA from two molecules of acetyl-CoA. *ACAT1* is responsible for cholesterol homeostasis and maintain appropriate cholesterol availability in cell membranes, whereas *ACACB* is the key regulator of the fatty acid oxidation pathways [40]. It controls fatty acid oxidation by means of the ability of malonyl-CoA to inhibit carnitine-palmitoyl-CoA transferase I (CPT1B). As in our work, Shige et al. [41] showed that vitamin E reduced the uptake of modified low-density lipoprotein (LDL) and suppressed ACAT activity, resulting in less cholesterol esterification in macrophages. Interestingly enough *ABCC4*, an ATP-binding cassette (ABC) transporter, *AKR7A2* which is involved in the detoxification of aldehydes and ketones, and finally *RSC1A1* which transport carbohydrate across the plasma membrane, were all down- regulated with VE treatment. In hepatocytes, ABCC4 was shown to be induced by oxidative stress through binding of the oxidative sensor nuclear factor E2-related factor 2 (Nrf2) to antioxidant-responsive element sequences in the promoter of *ABCC4* [42]. Our results showed for the first time that vitamin E down-regulates *ABCC4* expression. Because of significant differences in ADG between treatments, we validated the expression results of the *CISH*, *ABCC4*, *ACAT1* and *IGFR1* genes by qPCR, including in the statistical model ADG as a covariate. ADG was not significant, with a similar FC between the microarray and qPCR results (*r* ^2^ = 0.99; *P* = 0.008). Therefore, treatment was the main effect over gene expression.

In addition, we found that the transcription factors *FHL3*, *ZNF777* and *MAFB* were down-regulated. Considering all together, our results showed that supplementation with VE increased the content of α-tocopherol in the LT muscle and decreased metmyoglobin formation and lipid oxidation. We speculate that α-tocopherol in the LT muscle causes a decrease in the catalytic activity of enzymes involved in cellular transport of fatty acids and carbohydrates, and fatty acid oxidation in the mitochondria. Decreased *IGF1R* expression might be related to the lower lipid oxidation levels in VE animals. We also found that *IGFR1*, *ABCC4 ACAT1*, *CISH*, *ACACB*, *MYLK2*, *ZNF777* and *MAFB* were positively correlated with each other and with the diet, which suggest co-expression processes of these genes. We speculate that transcription factors *ZNF777*, *MAFB* and *FHL3* could be important players in mediating the effects of VE in regulating the expression of these genes.

To establish whether *IGFR1*, *ABCC4* and *ACAT1*, *CISH* are markers of meat oxidation or indirect markers of meat quality, further studies with greater numbers of animals are necessary.

A most dramatic effect of VE was observed on SF gene expression. *ALG11*, *SRPBR*, *DDX47*, *SEC23ID* and *TTC37* were among the most important genes in discriminate fed treatments. These genes were up-regulated and positively correlated with each other and with the diet, which might suggest co-expression processes. Four of the up-regulated genes in the VE group were related to heat shock proteins (HSPs) or chaperonin activity (*TTC37*, *DNAJC16*, *HSB8*, *AHSA1*). Some HSPs are characterized by various specific functions such as anti-apoptotic or anti-inflammatory effects [43]. In our study, these genes were up-regulated and positively correlated, suggesting putative increased stress protection in VE lambs.

Surprisingly VE treatment showed general up-regulation of almost all significant genes, compared to CON treatment. Lipid biosynthetic processes were among the most enriched functional clusters with major biological significance and importance (*PGAP3*, *EBP*, *CRLS1*, *MVD*, *CYP51A1*, *GNE*, *HMGCS1*, *DPAGT1*, *LSS*, *SIGMAR1*, *LPCAT3*, *FDFT1*, *DOLK*, *PIGM*, *SQLE*, *DHCR7*, *AGPAT9*, *LTA4H*, *PCYT2*, and *HSD17B7*). Moreover, genes implicated in sterol, steroid and cholesterol biosynthesis processes (*SREBF1*, *EBP*, *LDLR*, *MVD*, *CYP51A1*, *SQLE*, *DHCR7*, *INSIG1*, *HMGCS1*, *LSS*, and *FDFT1*) were up-regulated in the SF of VE animals, compared to the CON group. It has been previously reported that tocopherols inhibited *de novo* cholesterol synthesis within enterocytes [44], and cause repression of genes (*DHCR7* and *HMGCS1*) involved in the *de novo* synthesis of cholesterol in testes and adrenal glands [45]. The differences reported in previous studies and ours might be due to the different tissues analyzed. This supports even more our idea that there is a tissue specific response in response to VE supplementation. On the other hand, Wang et al. [46] found oxysterol-specific repressive effects in the *CYP51A1* and *FDFT1* genes, mediated via direct binding of the ligands to liver X receptor (LXR). Because of α–tocopherol and other antioxidants can inhibit the oxidation of cholesterol [47], the VE group could have a lower quantity of oxysterols and then have inhibited the repression of cholesterol biosynthesis via LXR and oxysterols and up-regulated the sterol, steroid and cholesterol biosynthesis processes. In this sense, several genes implicated in “the stress response” (*SOD3*, *IER3*, *HMGB2*, *UACA*, *LUC7L3*) were down-regulated in the VE group, compared to the CON group (Table 3 and Supplementary Table S1). Among them, *SOD3* showed higher values of FC. This gene encodes a member of the superoxide dismutase (SOD) protein family that protects the extracellular space from the toxic effects of reactive oxygen intermediates by converting superoxide radicals into hydrogen peroxide and oxygen. This finding contradicts the results of previous studies, which suggested that grass-based treatments elevated the activity of antioxidants, such as glutathione and superoxide dismutase (SOD), compared to grain-feeding [1]. However, Kumar et al. [48] in the myocardium muscle and Strobel et al. [49] in the skeletal muscle of exercise-trained and sedentary rats, found that antioxidant supplements reduced the endogenous antioxidants *SOD2* gene and protein and the glutathione peroxidase (GPx) gene and enzyme activity.

Another down-regulated gene in VE treatment was *IER3*. IER3-deficient NCM460 cells exhibited reduced reactive oxygen species levels, indicating increased antioxidative protection [50]. In our study, *IER3* was down regulated in the VE group suggesting an increase in antioxidative protection. The role of *HMGB1* in recognizing aberrant or damaged DNA has been shown in multiple in vitro experiments. A recent study directly showed the accumulation of HMGB1 at sites of oxidative DNA damage in live cells, thus defining HMGB1 as a component of an early DNA damage response [51]. A similar function has been attributed to HMGB2 [52]. These authors hypothesized that HMGB1/2 proteins act as a sensor of DNA modification, and their interaction with chemically altered DNA changes the chromatin structure, thus inducing DNA damage responses.

Finally, a cluster related to tRNA metabolic processes was also significant (*TRNT1*, *METTL1*, *ADAT2*, *NSUN2*, *IARS*, *GARS*, *EPRS* and *MARS*). Aminoacyl-tRNA synthetases perform an essential function in protein synthesis by catalyzing the esterification of an amino acid to its cognate tRNA (*IARS*, *GARS*, *EPRS* and *MARS*). Considering all together, we speculate that in the SF, vitamin E exerts anti-inflammatory effect and stress protection by increasing heat shock protein expression. Vitamin E reduces stress response probably as results of reduced reactive oxygen species levels. In addition animals supplemented with VE might have inhibited cholesterol oxidation in SF and enhanced sterol, steroid and cholesterol biosynthesis processes. And finally, we speculate that regulation of these gene expressions it could be mediated through tRNAs *IARS*, *GARS*, *EPRS* and *MARS*.

Regarding LT, we included ADG values as a covariate in the statistical model used to validate the expression differences by qPCR to avoid estimation biases. In this case, ADG was not significant, and a similar FC between microarray and qPCR results was found (*r* ^2^ = 0.99; *p* < 0.0001). Thus, the treatment was also the main effect over gene expression of SF.

Although we found differences in mRNA activity, it did not necessarily cause differences in metabolic processes. An increase in gene expression is not necessarily correlated with an increase in protein concentrations or enzyme activities. There are many processes between transcription and translation, including post-transcriptional, translational and protein degradation regulation, in controlling steady-state protein abundances [53]. Moreover, DNA microarray has been the technology of choice for transcriptome analysis in recent years. Nonetheless, array technology has several limitations which include: using microarray technology limits the researcher to detecting transcripts that correspond to existing genomic sequencing information; and background hybridization limits the accuracy of expression measurements, particularly for transcripts present in low abundance.

## Conclusion

This study demonstrated the beneficial effects of vitamin E supplementation during fattening period in lambs by increasing α-tocopherol content in the LT muscle and diminishing drastically the lipid oxidation of the meat. We observed a tissue-specific response to vitamin E supplementation. The gene expression profiles for VE and CON treatments were different in both LT and SF. Vitamin E supplementation had a dramatic effect on subcutaneous fat gene expression, showing a general up-regulation of genes, compared to CON treatment. Our study enabled us to identify novel genes (for example, *IGF1R*, *ACAT1*, *ABCC4*, *ACACB*, *SOD3*, and *IER3*) and metabolic pathways related to vitamin E metabolism that might be implicated in meat quality. To the best of our knowledge, this study was the first to report the effect of vitamin E supplementation on gene expression in the LT muscle and SF of lambs. Future exploration of these genes is necessary for a better understanding of how vitamin E affects the oxidative processes that occur in meat products.

## Methods

### Ethics statement

All experimental procedures including the care of animals and euthanasia were performed in accordance with the guidelines of the European Union and with Spanish regulations for the use and care of animals in research and were approved by the Animal Welfare Committee of the Centro de Investigación y Tecnología agroalimentaria (CITA) (protocol number 2009-01_MJT). In all cases, euthanasia was performed by penetrating captive bolt followed by immediate exsanguination.

### Animals and sample collection

Fourteen single reared male lambs of the Rasa Aragonesa breed were weaned at 48.28 ± 0.85 days of age. Seven lambs were fed a basal concentrate from weaning to slaughter (CON treatment, 24 ± 2.62 days). The remaining 7 lambs received for 4.71 ± 2.62 days the same basal concentrate as the CON group, and thereafter until slaughter, they received a similar concentrate with the same characteristics but enriched with 500 mg dl-α-tocopheryl acetate per kg of feed (VE treatment) for 33.28 ± 1.07 days. The ingredients and chemical composition of the feedstuffs are shown in Table 6. Prior to weaning, the CON and VE lambs suckled their mothers and had free access to their concentrates. The average concentrate intake of the CON and VE lambs during the experimental period was 24.3 and 25.1 kg per lamb, respectively. The experimental procedures, management of the animals and sample details for each group are described in detail in Ripoll et al. [11].

**Table 6:** Ingredients and chemical composition of the feedstuffs used in the experiment

Item	CON concentrate	VE concentrate^a^
Ingredients (%)		
Barley	40.87	40.87
Corn	14.95	14.95
Wheat	20.08	20.08
Soyabean meal	19.78	19.78
Salt	0.39	0.39
Carbonate	1.64	1.64
Mineral-vitamin mixture	1.20	1.10
Fat	1	1
Chemical composition		
CP, g/kg DM	175	175
NDF, g/kg DM	180	180
ADF, g/kg DM	45	45
α-tocopherol, mg/kg DM	27^b^	480^b^

DM, dry matter; CP, crude protein; NDF, neutral detergent fibre; ADF, acid detergent fibre

^a^Commercial concentrate enriched with vitamin E (Corderos Alendi Vitamina E-500, Ars Alendi S.A, Huesca, Spain). ^b^as mg dl-α-tocopheryl acetate/kg DM

All of the lambs were slaughtered when they attained 22–24 kg of slaughter weight (SW), according to the specifications of Ternasco de Aragón Protected Geographical Indication (Regulation (EC) No. 1107/96), which stipulates that lambs must be less than 90 days old with a SW between 22 and 24 kg. The lambs were slaughtered using EU laws in the same commercial abattoir, and the carcasses were hung by the Achilles tendon and chilled for 24 h at 4 °C in total darkness. The slaughter age, slaughter weight, and growth rate of the 2 management strategies are presented in Table 1.

Immediately after slaughter, a piece of LT muscle from the 12^th^ thoracic *vertebra* and a piece of SF between the atlas and axis cervical *vertebrae* were cut, frozen in liquid nitrogen and stored at−80 °C until RNA isolation.

### Analysis of α-tocopherol, intramuscular fat content, TBARS and metmyoglobin formation

After chilling, a piece of the LT muscle between the 4^th^ and the 6^th^ lumbar *vertebrae* was vacuum-packed and kept at−20 °C in darkness until the α-tocopherol analysis. The α-tocopherol concentration was determined by liquid extraction as described in González-Calvo et al. [54]. A portion of the loin between the 7^th^ and the 13^th^ thoracic *vertebrae* was used to measure the LT muscle oxidative processes. The color (metmyoglobin content, MMb) and lipid oxidation analysis (thiobarbituric acid-reactive substance, TBARS) were quantified after 7 days of being maintained in darkness at 4 °C. The LT muscle color and LT intramuscular fat oxidation were measured as described in González-Calvo et al. [54]. Briefly, the relative content of metmyoglobin (MMb) was estimated by the K/S_572/525_ ratio [55]. This ratio decreases when the MMb content increases. Intramuscular fat oxidation of the M. *Longissimus thoracis* was determined using the procedure reported by Pfalzgraf et al. [56]. The TBARS values are expressed as milligrams of malonaldehyde (MDA) kilogram^−1^ of muscle.

### RNA isolation and assessment of RNA integrity

Total RNA was extracted from approximately 500 mg of LT muscle or SF using RNeasy Tissue mini kits (QIAGEN, Madrid, Spain), following the manufacturer’s protocol. Prior to microarray analysis, RNA integrity and quality were assessed by a RNA 6000 Nano LabChip on the Agilent 2100 Bioanalyzer (Agilent, Madrid, Spain) and were quantified using a nanophotometric spectrophotometer (Implen, Madrid, Spain). All RNA integrity number (RIN) values were greater than 8.

### Microarray hybridization and data processing

RNA samples (*n* = 14, 7 samples from each treatment) were analyzed using Ovine Gene 1.1 ST Array Strip (Affymetrix, High Wycombe, UK). The Ovine gene 1.1 ST Array Strip allows for processing of four samples in parallel. This array contains a collection of 508538 probes that interrogate up to 26 unique sequences of each transcript (median probes/transcript = 23). These probes correspond to 22047 ovine genes. This study was part of a larger study in which we analyzed 84 samples corresponding to 3 treatments and 4 tissues (7 animals per treatment). In each strip, we hybridized the four tissues, selecting the animal tissue for each strip by random sampling. Microarray hybridization and scanning were performed at the Functional Genomics Core facility (Institute for Research in Biomedicine, IRB Barcelona, Spain), following the recommendations of the manufacturer. Scanned images (DAT files) were transformed into intensities (CEL files) by Affymetrix GeneChip Operating Software (GCOS). Overall array intensity was normalized between arrays to correct for systematic bias in the data and to remove the impact of non-biological influences on biological data. The imported data were analyzed at the gene-level, with exons summarized to genes, using the mean expression of all of the exons of a gene. Normalization was performed with the Robust Multi-Array Average (RMA) algorithm using quantile normalization, median polish probe summarization, and log2 probe transformation. The data sets supporting the results and discussed in this publication have been deposited in NCBI’s Gene Expression Omnibus repository [57] and are accessible through GEO Series accession number GSE63774 (https://www.ncbi.nlm.nih.gov/geo/query/acc.cgi?acc=GSE63774? acc = GSE63774).

### Validation of microarray data by qPCR

One microgram of RNA from each sample were treated with DNAse (Invitrogen, Carlsbad, CA, USA), and single-stranded cDNA was synthesized using the SuperScript®III Reverse Transcriptase kit (Invitrogen, Carlsbad, CA, USA), following the manufacturer’s recommendations. Specific exon-spanning primers for genes were generated and confirmed for specificity using BLAST (National Center for Biotechnology Information: http://www.ncbi.nlm.nih.gov/BLAST/). Before performing the qPCR reactions,conventional PCR was performed for all of the genes to test the primers and to verify the amplified products. The PCR products were sequenced to confirm gene identity using an ABI Prism 3700 (Applied Biosystems) with standard protocols. Homology searches were performed with BLAST (National Center for Biotechnology Information: https://blast.ncbi.nlm.nih.gov/Blast.cgi) to confirm the identity of the amplified fragments. Quantitative reverse transcriptase polymerase chain reaction (qRT-PCR) was performed using SYBR Green Master Mix: SYBR Premix Ex Taq II (Tli RNase H Plus) and an ABI Prism 7500 platform (Applied Biosystem, Madrid, Spain). Standard curves for each gene were generated to calculate the amplification efficiency through 4-fold serial dilution of cDNA pooled from the LT muscle and SF. The efficiency of PCR amplification for each gene was calculated using the standard curve method (*E* = 10^(−1/slope)^). Two “connector samples” were replicated in all of the plates to remove technical variation from this source of variability. The annealing temperatures, primer concentrations, and primer sequences for *CISH*, *ABCC4*, *ACAT1*, *IGF1R*, *LDLR*, *SQLE*, *SREBF1*, *SOD3*, *HAX1*, *HMGB2*, *METTL1*, *HSPB8*, and *IER3* (genes of interest: GOIs) and the reference genes (*GUSB* and *YWHAZ*) are described in Table 7. These reference genes were chosen because in previous studies they have been shown to be the most stable in these tissues [5].

**Table 7:** Primers forward and reverse used in RT-PCR

PCR conditions^a^							
Symbol	Gene	Primers forward and reverse	Amplicon bp	AT	nM	E	*R* ^2^
*CISH*	Cytokine inducible SH2-containing protein	F:5′-agccaagaccttctcctacct-3′	96	60	600/600	1.98	0.99
		R:5′-acgaggaagacagtgaagacg-3′					
*ABCC4*	ATP-binding cassette, sub-family C (CFTR/MRP), member 4	F: 5′-ccgtgagaaatttgcccactg-3′	128	60	900/900	2.00	0.99
		R: 5′- gcaaaacatacggctcatcat-3′					
*ACAT1*	A cetyl-Coenzyme A acetyltransferase 1	F: 5′-gagctgtttctcttggacatcc-3′	118	60	900/900	1.98	0.99
		R: 5′- cctcctcctccgttgcaaat-3′					
*IGF1R*	Insulin-like growth factor 1 receptor	F: 5′-ccaaggcctgagaactccat-3′	122	60	900/900	2.00	0.98
		R: 5′- ctggacacacattcccgct-3′					
*LDLR*	Low density lipoprotein receptor	F:5′- gcatcaacttcgacaaccct -3′	112	60	600/600	2.00	0.98
		R:5′- tcctccaagctgaccatctg -3′					
*SQLE*	Squalene epoxidase	F:5′-aatgtgttgcaggtccggtt-3′	88	60	600/600	1.98	0.98
		R:5′- tagactgcgacgccaaagaa -3′					
*SREBF1*	Sterol regulatory element binding transcription factor 1	F:5′- ctgctatgcaggcagcac -3′	99	60	200/200	2.00	0.98
		R:5′- ggttgatgggcagcttgt -3′					
*SOD3*	Superoxide dismutase 3, extracellular	F:5′-atccacgtgcaccagtttg-3′	110	60	300/300	2.00	0.98
		R:5′-aagttgccaaagtcgccc-3′					
*HAX1*	HCLS1 associated protein X-1	F:5′-taacccatcaagaggcaggc-3′	115	60	600/600	1.99	0.99
		R:5′-gaccggaaccaacgtcctag-3′					
*HMGB2*	High-mobility group box 2	F:5′-gccgtatgaacagaaagcagc-3′	126	60	600/600	1.94	0.99
		R:5′-ttcttcttggagcctgtcgg-3′					
*HSPB8*	Heat shock 22 kDa protein 8	F:5′-agcaagaaggtggcatcgtt-3′	126	60	400/400	2.00	0.99
		R:5′- cctggggagcttcgatgatc-3′					
*IER3*	Immediate early response 3	F:5′-ttcaccttcgaccctctccc-3′	130	60	400/400	1.90	0.97
		R:5′- cctcgactggcagctgac-3′					
*METTL1*	Methyltransferase like 1	F:5′-ggagctgcatgagtggatgt-3′	104	60	600/600	1.97	0.99
		R:5′- tgcccagatgtcccacaatg-3′					
*GUSB*	β-glucuronidase	F:5′-gcttcgagcagcagtggta-3′	86	60	600/600	1.99	0.99
		R:5′-cacgtcgttgaagctggac-3′					
*YWHAZ*	Tyrosine 3-monooxygenase/tryptophan 5-monooxygenase activation protein	F:5′- tgtaggagcccgtaggtcatct-3′	102	60	400/400	1.99	0.98
		R:5′ - ttctctctgtattctcgagccatct-3					

^a^Real-time PCR conditions: annealing temperature (AT), primer concentrations (nM), E: primer efficiency and *R* ^2^: correlation coefficient

### Statistical analysis

#### Statistical analysis of performance, α-tocopherol and lipid oxidation in the LT muscle

Statistical analysis was performed using a general lineal model (GLM). Treatment (CON and VE) was included as the fixed factor for weaning age (WA), slaughter age (SA), slaughter weight, intramuscular fat content (IMF) and average daily gain (ADG). For muscle α-tocopherol, myoglobin and TBARS, the model also included intramuscular fat content and slaughter age as covariates. The results are expressed as least square means ± the standard error (SE) values and the differences were tested at a level of significance of 0.05 with the t statistic.

#### Statistical analysis for the identification of differentially expressed genes by microarray analysis in LT and SF

Normalized data were further analyzed using Babelomics (http://babelomics.bioinfo.cipf.es/) and MetaboAnalyst software [58]. Genes showing a statistically significant value of the Limma-test; (*P* < 0.01) were screened out as differentially expressed between the treatments. Significant genes were annotated based on similarity scores in blastn comparisons of Affymetrix Transcript cluster sequences against ovine sequences in GenBank. In addition, a second method, significance analysis of microarray (SAM), was used to identify and reconfirm differentially expressed genes in VE-CON contrasts. Furthermore, SAM was used to detect false positive significant genes from Limma-testing. SAM is a well-established statistical method for the identification of differentially expressed genes in microarray data analysis [58]. SAM is designed to address the FDR when running multiple tests on high-dimensional microarray data. First, it assigns a significance score to each variable based on its relative change from the standard deviation of repeated measurements. Then, it chooses variables with scores greater than an adjustable threshold Δ and compares their relative differences from the distribution estimated by random permutations of the class labels. For each Δ, a certain proportion of the variables in the permutation set will be found to be significant by chance. This number is used to calculate the FDR. Underestimation of the variability inflates the value of the statistic and can result in an increased number of false positives.

#### Multivariate analysis of gene expression

Multivariate analysis was performed using MetaboAnalyst, according to Xia et al. [58]. Principal component analysis (PCA), partial least squares discriminate analysis (PLS-DA), and variable importance of projection (VIP) were used to cluster the samples based on the selected gene expression profiles. Principal component analysis was used to reduce the large set of variables (genes) into 2 principal components (PCA 1 and 2). PLS-DA was used to enhance the separation between the groups by summarizing the data into a few latent variables that maximized covariance between the response and the predictors. The corresponding loading plot was used to determine the genes most responsible for separation in the PLS-DA score plot. Based on the PLS-DA results, genes were plotted according to their importance in separating the dietary groups and each gene received a value called the variable importance in the projection. Variable importance in the projection values >1 suggests that the variable is significantly involved in the separation of groups [59]. Variables with the highest VIP values were the most powerful group of discriminators. We also investigated trends or patterns in gene expression changes.

#### Hierarchical clustering analysis (HCA)

Cluster analysis was performed using MetaboAnalyst. In (agglomerative) hierarchical cluster analysis, each sample begins as a separate cluster and the algorithm proceeds to combine them until all of the samples belong to one cluster. Two parameters must be considered when performing hierarchical clustering. The first is the similarity measure, and the other is the clustering algorithm. For distance measurements we used the Euclidian and Ward algorithm for clustering. The results are shown as a heatmap.

#### Statistical analysis of gene expression validated by qPCR

The corresponding mRNA levels were measured and analyzed by their quantification cycles (Cq). The statistical methods to analyze differences in the expression rate were performed following the method proposed by Steibel et al. [60]. The mixed model fitted was:

$$ {{\mathrm{y}}_{\mathrm{rigkm}}}_{=}\mathrm{T}{\mathrm{G}}_{\mathrm{gi}} + \mathrm{b}{\left(\mathrm{I}\mathrm{M}\mathrm{F}\right)}_{\mathrm{m}} + \mathrm{b}\left(\mathrm{A}\mathrm{D}\mathrm{G}\right)\mathrm{m} + {\mathrm{P}}_{\mathrm{k}} + {\mathrm{A}}_{\mathrm{m}} + {\mathrm{e}}_{\mathrm{rigkm}} $$

where, *y*_***rigkm***_ is the *C*_q_ value (transformed data taking into account *E* < 2) of the g^th^ gene (GOIs and housekeeping) from the r^th^ well in the k^th^ plate collected from the m^th^ animal subjected to the i^th^ treatment (CON, VE and ALF); TG_gi_ is the fixed interaction among the i^th^ treatment and the g^th^ gene; IMF_m_ (only used in *L. Thoracis* muscle tissue gene expression)_,_ and ADG_m_ are the effects of intramuscular fat, and the average daily gain of the m^th^ animal included as covariates; P_k_ is the fixed effect of the k^th^ plate; A_m_ is the random effect of the m^th^ animal from which samples were collected (A_m_ ~ (0,σ^2^_A_)); and e_rigkm_ is the random residual. Gene specific residual variance (heterogeneous residual) was fitted to the gene by treatment effect (*e*_***rigkm***_ ~ N (0, σ^2^_egi_).

To test differences (*diff*_*GOI*_) in the expression rates of the target genes between treatments in terms of fold changes (FCs), the approach suggested by Steibel et al. [60] was used. The significance of the *diff*_*GOI*_ estimates was determined with the t statistic.

### Functional annotation analyses

The Database for Annotation, Visualization and Integrated Discovery (DAVID) v6.7b [61], was used to determine the pathways and processes of major biological significance and importance through the Functional Annotation Cluster (FAC) tool based on the Gene Ontology (GO) annotation function. DAVID FAC analysis was performed with the gene lists obtained after SAM analysis. Medium stringency EASE score parameters were selected to indicate confident enrichment scores of functional significance and the importance of the given pathways and processes investigated. An enrichment score of 1.3 was employed as a threshold for cluster significance [61].

## Additional files

Additional file 1: Table S1. The significant genes identified by SAM in VE vs. CON contrast. (DOCX 62 kb)
Additional file 2: Figure S1. Hierarchical clustering analysis in subcutaneous fat using 330 SAM genes. (DOCX 290 kb)
Additional file 3: Table S2. DAVID Functional Annotation Clustering of SAM genes in VE vs. CON muscle L. Thoracis. Only is shown the 2 most enrichment cluster. (DOCX 16 kb)
