# Supplementary material for: Genome-wide expression profiling in muscle and subcutaneous fat of lambs in response to the intake of concentrate supplemented with vitamin E
Source: BMC Genomics. 2017 Jan 17;18:92. doi: 10.1186/s12864-016-3405-8 (PMC5240399; doi:10.1186/s12864-016-3405-8)
Supplement: Additional file 1: Table S1. — The significant genes identified by SAM in VE vs. CON contrast. (DOCX 62 kb) [file 12864_2016_3405_MOESM1_ESM.docx]

| **Supplementary Table S1.**  **The significant genes identified by SAM in VE vs. CON contrast.** | | | |
| --- | --- | --- | --- |
| **Gene** | **Gene Name** | **qValue** | **FC** |
| **Down-regulated genes** | | |  |
| *SOD3* | superoxide dismutase 3, extracellular | 2.90E-05 | -2.78 |
| *CLEC3B* | C-type lectin domain family 3, member B | 9.43E-05 | -2.31 |
| *METTL7A* | methyltransferase like 7A | 2.83E-05 | -2.09 |
| *IER3* | immediate early response 3 | 2.81E-05 | -2.06 |
| *PLK2* | polo-like kinase 2 | 5.92E-05 | -1.99 |
| *CLEC1A* | C-type lectin domain family 1, member A | 5.39E-05 | -1.80 |
| *HSPA1A* | heat shock 70kDa protein 1A; heat shock 70kDa protein 1B | 4.23E-05 | -1.78 |
| *TNFSF10* | tumor necrosis factor (ligand) superfamily, member 10 | 0.000111 | -1.70 |
| *MERTK* | c-mer proto-oncogene tyrosine kinase | 8.18E-05 | -1.68 |
| *MLLT3* | myeloid/lymphoid or mixed-lineage leukemia; translocated to, 3 | 0.000112 | -1.67 |
| *TPPP2* | tubulin polymerization-promoting protein family member 2 | 2.90E-05 | -1.57 |
| *HMGB2* | high-mobility group box 2 | 6.66E-05 | -1.53 |
| *PECAM1* | platelet/endothelial cell adhesion molecule | 7.51E-05 | -1.49 |
| *IFITM3* | interferon induced transmembrane protein 3 | 8.47E-05 | -1.47 |
| *H1F0* | H1 histone family, member 0 | 2.90E-05 | -1.46 |
| *HMG20B* | high-mobility group 20B | 4.20E-05 | -1.45 |
| *GALK1* | galactokinase 1 | 4.44E-05 | -1.45 |
| *NFIL3* | naked cuticle homolog 1 | 5.04E-05 | -1.42 |
| *CDH5* | cadherin 5, type 2 (vascular endothelium) | 7.29E-05 | -1.42 |
| *ERG* | v-ets erythroblastosis virus E26 oncogene homolog | 9.43E-05 | -1.41 |
| *CEBPA* | CCAAT/enhancer binding protein (C/EBP), alpha | 8.13E-05 | -1.39 |
| *ANKRD44* | ankyrin repeat domain 44 | 4.72E-05 | -1.36 |
| *HCST* | hematopoietic cell signal transducer | 5.11E-05 | -1.34 |
| *UACA* | uveal autoantigen with coiled-coil domains and ankyrin repeats | 2.95E-05 | -1.34 |
| *IFITM1* | interferon induced transmembrane protein 1 | 5.70E-05 | -1.31 |
| *RPL10* | ribosomal protein L10; 9 | 0.000113 | -1.31 |
| *PID1* | phosphotyrosine interaction domain containing 1 | 7.29E-05 | -1.31 |
| *LUC7L3* | cisplatin resistance-associated overexpressed protein | 6.76E-05 | -1.30 |
| *H3F3A* | H3 histone, family 3B (H3.3B) | 8.18E-05 | -1.29 |
| *AMOTL2* | angiomotin like 2 | 2.81E-05 | -1.28 |
| *PSIP1* | PC4 and SFRS1 interacting protein 1 | 2.81E-05 | -1.28 |
| *RPL17* | ribosomal protein L17 pseudogene 22; | 2.81E-05 | -1.25 |
| *TRA2A* | transformer 2 alpha homolog | 0.000109 | -1.23 |
| *RPS25* | ribosomal protein S25 pseudogene 8; ribosomal protein S25 | 8.74E-05 | -1.16 |
| ***Up-regulated genes*** | | |  |
| *ZNF564* | zinc finger protein 564 | 5.56E-05 | 2.88 |
| *CYP51A1* | cytochrome P450, family 51, subfamily A, polypeptide 1 | 7.73E-05 | 2.80 |
| *SQLE* | squalene epoxidase | 3.91E-05 | 2.70 |
| *TMEM106C* | transmembrane protein 106C | 2.90E-05 | 2.70 |
| *SLC25A16* | solute carrier family 25 , member 16 | 2.81E-05 | 2.67 |
| *LDLR* | low density lipoprotein receptor | 2.81E-05 | 2.66 |
| *MVD* | mevalonate (diphospho) decarboxylase | 6.62E-05 | 2.56 |
| *SLC5A6* | solute carrier family 5 (sodium-dependent vitamin transporter), member 6 | 3.16E-05 | 2.50 |
| *SLC20A1* | solute carrier family 20 (phosphate transporter), member 1 | 2.90E-05 | 2.44 |
| *INSIG1* | insulin induced gene 1 | 2.90E-05 | 2.44 |
| *PCYT2* | phosphate cytidylyltransferase 2, ethanolamine | 4.26E-05 | 2.37 |
| *AGPAT9* | 1-acylglycerol-3-phosphate O-acyltransferase 9 | 7.77E-05 | 2.33 |
| *STARD4* | StAR-related lipid transfer (START) domain containing 4 | 4.61E-05 | 2.31 |
| *SRPRB* | signal recognition particle receptor, B subunit | 0 | 2.30 |
| *LPCAT3* | lysophosphatidylcholine acyltransferase 3 | 3.85E-05 | 2.28 |
| *TIMM8A* | translocase of inner mitochondrial membrane 8 homolog A | 2.81E-05 | 2.27 |
| *DLAT* | dihydrolipoamide S-acetyltransferase | 7.29E-05 | 2.26 |
| *HMGCS1* | 3-hydroxy-3-methylglutaryl-Coenzyme A synthase 1 (soluble) | 4.70E-05 | 2.18 |
| *DHCR7* | 7-dehydrocholesterol reductase | 3.77E-05 | 2.12 |
| *ENTPD1* | ectonucleoside triphosphate diphosphohydrolase 1 | 5.04E-05 | 2.11 |
|  |  |  |  |
| ***Continued*** |  |  |  |
| ***Gene*** | **Gene Name** | **qValue** | **FC** |
| *LTA4H* | leukotriene A4 hydrolase | 4.45E-05 | 2.10 |
| *PJA1* | praja ring finger 1 | 2.81E-05 | 2.08 |
| *METTL13* | methyltransferase like 13 | 7.77E-05 | 2.06 |
| *MTFP1* | mitochondrial fission process 1 | 7.46E-05 | 2.06 |
| *PANK1* | pantothenate kinase 1 | 2.81E-05 | 2.03 |
| *TFRC* | transferrin receptor (p90, CD71) | 2.81E-05 | 1.97 |
| *TOMM40* | translocase of outer mitochondrial membrane 40 homolog | 2.81E-05 | 1.96 |
| *FAM49B* | family with sequence similarity 49, member B | 3.05E-05 | 1.96 |
| *PEX2* | peroxisomal membrane protein 3, 35kDa | 2.81E-05 | 1.95 |
| *RDH11* | retinol dehydrogenase 11 (all-trans/9-cis/11-cis) | 2.83E-05 | 1.95 |
| *ACAT2* | acetyl-Coenzyme A acetyltransferase 2 | 6.16E-05 | 1.93 |
| *RPS4Y1* | ribosomal protein S4, Y-linked 1 | 2.81E-05 | 1.90 |
| *SLC48A1* | solute carrier family 48 (heme transporter), member 1 | 3.91E-05 | 1.89 |
| *ZMYM6NB* | ZMYM6 neighbor | 5.04E-05 | 1.88 |
| *MRPS7* | mitochondrial ribosomal protein S7 | 6.49E-05 | 1.87 |
| *HSPB8* | heat shock 22kDa protein 8 | 4.30E-05 | 1.87 |
| *TMEM37* | transmembrane protein 37 | 2.90E-05 | 1.87 |
| *PPARGC1B* | peroxisome proliferator-activated receptor gamma, coactivator 1 beta | 2.81E-05 | 1.86 |
| *DOLK* | dolichol kinase | 2.81E-05 | 1.86 |
| *TMEM68* | transmembrane protein 68 | 2.81E-05 | 1.85 |
| *HSPH1* | heat shock 105kDa/110kDa protein 1 | 4.98E-05 | 1.84 |
| *C29H11orf54* | chromosome 11 open reading frame 54 | 7.49E-05 | 1.83 |
| *DCTN5* | dynactin 5 (p25) | 3.91E-05 | 1.82 |
| *IDH3A* | isocitrate dehydrogenase 3 (NAD+) alpha | 5.04E-05 | 1.82 |
| *OXNAD1* | prolyl 4-hydroxylase, alpha polypeptide I | 4.53E-05 | 1.82 |
| *CRLS1* | cardiolipin synthase 1 | 6.56E-05 | 1.81 |
| *EEF1E1* | eukaryotic translation elongation factor 1 epsilon 1 | 2.81E-05 | 1.81 |
| *MRPL51* | mitochondrial ribosomal protein L51 | 7.51E-05 | 1.80 |
| *MTX2* | metaxin 2 | 4.75E-05 | 1.80 |
| *DOLPP1* | dolichyl pyrophosphate phosphatase 1 | 2.81E-05 | 1.79 |
| *HSPA13* | heat shock protein 70kDa family, member 13 | 5.04E-05 | 1.78 |
| *ABCF3* | ATP-binding cassette, sub-family F (GCN20), member 3 | 2.81E-05 | 1.78 |
| *EGLN3* | egl nine homolog 3 (C. elegans) | 7.49E-05 | 1.76 |
| *SREBF1* | sterol regulatory element binding transcription factor 1 | 4.44E-05 | 1.76 |
| *CERS6* | ceramide synthase 6 | 2.81E-05 | 1.76 |
| *MRPL15* | mitochondrial ribosomal protein L15 | 7.22E-05 | 1.76 |
| *ADPGK* | ADP-dependent glucokinase | 2.81E-05 | 1.75 |
| *NDUFS6* | nuclear factor, interleukin 3 regulated | 4.70E-05 | 1.74 |
| *EIF2B1* | eukaryotic translation initiation factor 2B, subunit 1 alpha, 26kDa | 2.81E-05 | 1.73 |
| *RWDD2B* | RWD domain containing 2B | 3.16E-05 | 1.73 |
| *LSS* | lanosterol synthase (2,3-oxidosqualene-lanosterol cyclase) | 2.81E-05 | 1.72 |
| *EIF1AD* | eukaryotic translation initiation factor 1A domain containing | 2.90E-05 | 1.72 |
| *DPP3* | dipeptidyl-peptidase 3 | 3.04E-05 | 1.71 |
| *FAM210A* | family with sequence similarity 210, member A | 2.81E-05 | 1.71 |
| *PTCD2* | pentatricopeptide repeat domain 2 | 6.16E-05 | 1.70 |
| *AMD1* | adenosylmethionine decarboxylase 1 | 2.81E-05 | 1.70 |
| *PPIL1* | peptidylprolyl isomerase (cyclophilin)-like 1 | 2.81E-05 | 1.70 |
| *DPH3* | DPH3, KTI11 homolog (S. cerevisiae); DPH3B, KTI11 homolog B | 2.81E-05 | 1.70 |
| *KCTD14* | potassium channel tetramerisation domain containing 14 | 7.55E-05 | 1.69 |
| *EFNB1* | ephrin-B1 | 2.90E-05 | 1.69 |
| *TAF9* | TAF9 RNA polymerase II, | 4.01E-05 | 1.69 |
| *ABCE1* | similar to ATP-binding cassette, sub-family E, member 1 | 1.53E-05 | 1.69 |
| *PINX1* | PIN2-interacting protein 1 | 2.81E-05 | 1.69 |
| *DNM1L* | dynamin 1-like | 3.05E-05 | 1.68 |
| *GARS* | glycyl-tRNA synthetase | 7.77E-05 | 1.68 |
| *LTV1* | similar to putative protein STRF7; LTV1 homolog | 4.98E-05 | 1.68 |
| *EBP* | emopamil binding protein (sterol isomerase) | 8.13E-05 | 1.67 |
| ***Continued*** |  |  |  |
| ***Gene*** | **Gene Name** | **qValue** | **FC** |
| *ACTR1A* | ARP1 actin-related protein 1 homolog A, centractin alpha | 2.90E-05 | 1.67 |
| *C5H22orf28* | RNA 2',3'-Cyclic Phosphate And 5'-OH Ligase | 2.81E-05 | 1.67 |
| *MRPS18B* | mitochondrial ribosomal protein S18B | 5.04E-05 | 1.67 |
| *ALG11* | UTP14, U3 small nucleolar ribonucleoprotein, homolog C | 0 | 1.67 |
| *ALDH18A1* | aldehyde dehydrogenase 18 family, member A1 | 2.81E-05 | 1.67 |
| *RAC3* | ras-related C3 botulinum toxin substrate 3 | 2.81E-05 | 1.66 |
| *PRMT5* | protein arginine methyltransferase 5 | 6.01E-05 | 1.66 |
| *GTF3C6* | general transcription factor IIIC, polypeptide 6, alpha 35kDa | 5.62E-05 | 1.66 |
| *SENP8* | SUMO/sentrin specific peptidase family member 8 | 5.62E-05 | 1.66 |
| *ABCF2* | ATP-binding cassette, sub-family F (GCN20), member 2 | 4.70E-05 | 1.65 |
| *TMEM181* | transmembrane protein 181 | 1.07E-05 | 1.65 |
| *NDUFAF4* | NADH dehydrogenase (ubiquinone) 1 alpha subcomplex, assembly factor 4 | 7.29E-05 | 1.65 |
| *WRB* | tryptophan rich basic protein | 2.90E-05 | 1.65 |
| *ZW10* | ZW10, kinetochore associated, homolog | 2.81E-05 | 1.65 |
| *PLEKHA8* | pleckstrin homology domain containing | 2.81E-05 | 1.65 |
| *KPNA6* | karyopherin alpha 6 (importin alpha 7) | 2.81E-05 | 1.65 |
| *ABHD8* | abhydrolase domain containing 8 | 5.60E-05 | 1.64 |
| *SLC16A1* | solute carrier family 16, member 1 | 5.62E-05 | 1.64 |
| *SIGMAR1* | sigma non-opioid intracellular receptor 1 | 4.44E-05 | 1.64 |
| *SRP54* | similar to signal recognition particle 54kDa | 3.95E-05 | 1.64 |
| *NUS1* | oxidoreductase NAD-binding domain containing 1 | 2.90E-05 | 1.64 |
| *CAV2* | caveolin 2 | 7.49E-05 | 1.63 |
| *AKIP1* | A Kinase (PRKA) Interacting Protein 1 | 2.81E-05 | 1.63 |
| *EXTL3* | exostoses (multiple)-like 3 | 2.04E-05 | 1.63 |
| *LEO1* | Leo1, Paf1/RNA polymerase II complex component, homolog | 2.81E-05 | 1.62 |
| *PROSC* | proline synthetase co-transcribed homolog | 2.81E-05 | 1.62 |
| *PDRG1* | p53 and DNA-damage regulated 1 | 6.66E-05 | 1.62 |
| *HSD17B7* | hydroxysteroid (17-beta) dehydrogenase 7 | 3.04E-05 | 1.62 |
| *GFER* | growth factor, augmenter of liver regeneration | 3.59E-05 | 1.62 |
| *MRS2* | MRS2 magnesium homeostasis factor homolog | 2.81E-05 | 1.62 |
| *ATP6AP2* | ATPase, H+ transporting, lysosomal accessory protein 2 | 2.81E-05 | 1.62 |
| *SEC23IP* | SEC23 interacting protein | 1.07E-05 | 1.61 |
| *VIMP* | VCP-interacting membrane protein | 7.46E-05 | 1.60 |
| *EMC8* | ER membrane protein complex subunit 8 | 2.81E-05 | 1.59 |
| *UBA2* | ubiquitin-like modifier activating enzyme 2 | 3.91E-05 | 1.58 |
| *ADSS* | adenylosuccinate synthase | 2.47E-05 | 1.58 |
| *PTCD3* | Pentatricopeptide repeat domain 3 | 4.16E-05 | 1.58 |
| *VMP1* | vacuole membrane protein 1 | 2.90E-05 | 1.58 |
| *MAOB* | monoamine oxidase B | 7.77E-05 | 1.58 |
| *THUMPD3* | THUMP domain containing 3 | 5.04E-05 | 1.58 |
| *SLC39A3* | solute carrier family 39 (zinc transporter), member 3 | 2.81E-05 | 1.57 |
| *ARMC1* | armadillo repeat containing 1 | 3.05E-05 | 1.57 |
| *TRAPPC3* | trafficking protein particle complex 3 | 2.81E-05 | 1.57 |
| *SLC31A1* | solute carrier family 31 (copper transporters), member 1 | 5.11E-05 | 1.56 |
| *MRPL12* | mitochondrial ribosomal protein L12 | 8.13E-05 | 1.56 |
| *MARS* | methionyl-tRNA synthetase | 2.23E-05 | 1.56 |
| *ELP3* | elongation protein 3 homolog (S. cerevisiae) | 1.07E-05 | 1.56 |
| *TIMM22* | translocase of inner mitochondrial membrane 22 homolog | 2.04E-05 | 1.56 |
| *USP5* | ubiquitin specific peptidase 5 (isopeptidase T) | 2.90E-05 | 1.55 |
| *RASSF8* | Ras association (RalGDS/AF-6) domain family (N-terminal) member 8 | 1.07E-05 | 1.55 |
| *STRAP* | serine/threonine kinase receptor associated protein | 3.04E-05 | 1.55 |
| *UBA5* | ubiquitin-like modifier activating enzyme 5 | 2.83E-05 | 1.55 |
| *GPN1* | GPN-loop GTPase 1 | 5.04E-05 | 1.55 |
| *CNIH* | cornichon homolog | 2.81E-05 | 1.55 |
| *KPNA1* | karyopherin alpha 1 (importin alpha 5) | 2.81E-05 | 1.54 |
| *DRG1* | developmentally regulated GTP binding protein 1 | 3.04E-05 | 1.54 |
| *POGLUT1* | protein O-glucosyltransferase 1 | 2.81E-05 | 1.53 |
| ***Continued*** |  |  |  |
| ***Gene*** | **Gene Name** | **qValue** | **FC** |
| *RIOK2* | RIO kinase 2 | 2.81E-05 | 1.53 |
| *CXorf56* | chromosome X open reading frame 56 | 2.81E-05 | 1.53 |
| *ABHD6* | abhydrolase domain containing 6 | 5.00E-05 | 1.53 |
| *MCU* | mitochondrial calcium uniporter | 2.81E-05 | 1.53 |
| *RNF26* | ring finger protein 26 | 2.81E-05 | 1.53 |
| *PPP2R4* | protein phosphatase 2A activator, regulatory subunit 4 | 7.16E-05 | 1.53 |
| *VCL* | vinculin | 2.81E-05 | 1.53 |
| *METTL1* | methyltransferase like 1 | 7.95E-05 | 1.53 |
| *CMPK1* | cytidine monophosphate (UMP-CMP) kinase 1, cytosolic | 7.39E-05 | 1.53 |
| *PLA2G16* | phospholipase A2, group XVI | 2.81E-05 | 1.52 |
| *NOLC1* | NOP16 nucleolar protein homolog | 2.90E-05 | 1.52 |
| *CIAPIN1* | cytokine induced apoptosis inhibitor 1 | 6.56E-05 | 1.52 |
| *IRF8* | interferon regulatory factor 8 | 7.46E-05 | 1.52 |
| *TMEM242* | transmembrane protein 242 | 2.81E-05 | 1.52 |
| *DNAJC11* | DnaJ (Hsp40) homolog, subfamily C, member 11 | 4.98E-05 | 1.52 |
| *SNX19* | sorting nexin 19 | 2.81E-05 | 1.52 |
| *HNRNPH2* | ribosomal protein L36a pseudogene 51 | 2.81E-05 | 1.52 |
| *IDH1* | isocitrate dehydrogenase 1 (NADP+), soluble | 6.01E-05 | 1.52 |
| *TMEM203* | transmembrane protein 203 | 1.53E-05 | 1.52 |
| *RFT1* | RFT1 homolog | 5.02E-05 | 1.52 |
| *MKLN1* | muskelin 1, intracellular mediator containing kelch motifs | 2.81E-05 | 1.51 |
| *TSTA3* | tissue specific transplantation antigen P35B | 2.90E-05 | 1.51 |
| *TIMM44* | translocase of inner mitochondrial membrane 44 homolog | 7.73E-05 | 1.50 |
| *MTERFD1* | MTERF domain containing 1 | 2.90E-05 | 1.50 |
| *TSR1* | TSR1, 20S rRNA accumulation, homolog | 2.81E-05 | 1.50 |
| *VPS45* | vacuolar protein sorting 45 homolog | 7.73E-05 | 1.50 |
| *DDX47* | DEAD (Asp-Glu-Ala-Asp) box polypeptide 47 | 0 | 1.50 |
| *PBDC1* | polysaccharide biosynthesis domain containing 1 | 2.81E-05 | 1.50 |
| *MRPS35* | mitochondrial ribosomal protein S35 | 6.01E-05 | 1.50 |
| *SLC39A7* | solute carrier family 39 (zinc transporter), member 7 | 2.23E-05 | 1.49 |
| *NADK* | NAD kinase | 2.90E-05 | 1.49 |
| *SLC39A10* | solute carrier family 39 (zinc transporter), member 10 | 4.08E-05 | 1.49 |
| *ARL2BP* | ADP-ribosylation factor-like 2 binding protein | 3.04E-05 | 1.49 |
| *AKAP10* | A kinase (PRKA) anchor protein 10 | 2.81E-05 | 1.48 |
| *MTRF1L* | mitochondrial translational release factor 1-like | 5.56E-05 | 1.48 |
| *TGFBR1* | transforming growth factor, beta receptor 1 | 3.30E-05 | 1.48 |
| *P4HA1* | phosphatase domain containing, paladin 1 | 7.22E-05 | 1.48 |
| *NUP35* | nucleoporin 43kDa | 2.90E-05 | 1.48 |
| *CNPY4* | canopy 4 homolog | 2.81E-05 | 1.48 |
| *FXC1* | fracture callus 1 homolog | 3.04E-05 | 1.48 |
| *NKD1* | nucleolar and coiled-body phosphoprotein 1 | 3.91E-05 | 1.47 |
| *EIF2B2* | eukaryotic translation initiation factor 2B, subunit 2 beta, 39kDa | 2.81E-05 | 1.47 |
| *CXXC5* | CXXC finger 5 | 7.29E-05 | 1.47 |
| *FAM98A* | family with sequence similarity 98, member A | 2.81E-05 | 1.47 |
| *UHMK1* | U2AF homology motif (UHM) kinase 1 | 2.81E-05 | 1.47 |
| *TRNT1* | tRNA nucleotidyl transferase, CCA-adding, 1 | 6.96E-05 | 1.47 |
| *ERAL1* | Era G-protein-like 1 | 4.30E-05 | 1.47 |
| *LRRC59* | leucine rich repeat containing 59 | 1.07E-05 | 1.47 |
| *ANAPC7* | anaphase promoting complex subunit 7 | 2.81E-05 | 1.47 |
| *GFM2* | G elongation factor, mitochondrial 2 | 6.96E-05 | 1.47 |
| *ADPRH* | ADP-ribosylarginine hydrolase | 5.95E-05 | 1.47 |
| *SNRNP40* | small nuclear ribonucleoprotein 40kDa (U5) | 2.81E-05 | 1.46 |
| *DNAJA3* | DnaJ (Hsp40) homolog, subfamily A, member 3 | 2.81E-05 | 1.46 |
| *RER1* | RER1 retention in endoplasmic reticulum 1 homolog | 2.04E-05 | 1.46 |
| *UGGT1* | UDP-glucose ceramide glucosyltransferase-like 1 | 2.90E-05 | 1.46 |
| *POLR2E* | polymerase (RNA) II (DNA directed) polypeptide E, 25kDa | 2.90E-05 | 1.46 |
| *NUDT9* | nucleoporin 35kDa | 2.81E-05 | 1.46 |
| ***Continued*** |  |  |  |
| ***Gene*** | **Gene Name** | **qValue** | **FC** |
| *FTSJ2* | FtsJ homolog 2 | 2.90E-05 | 1.46 |
| *TMEM33* | transmembrane protein 33 | 5.04E-05 | 1.46 |
| *GSKIP* | GSK3B interacting protein | 2.90E-05 | 1.45 |
| *GNG7* | guanine nucleotide binding protein (G protein), gamma 7 | 4.01E-05 | 1.45 |
| *MAN1A2* | mannosidase, alpha, class 1A, member 2 | 4.68E-05 | 1.45 |
| *FBXO33* | F-box protein 33 | 2.81E-05 | 1.45 |
| *GMPS* | guanine monphosphate synthetase | 2.23E-05 | 1.45 |
| *HNRNPAB* | heterogeneous nuclear ribonucleoprotein A/B | 2.81E-05 | 1.45 |
| *MRPL48* | mitochondrial ribosomal protein L48 | 4.98E-05 | 1.45 |
| *PDE12* | phosphodiesterase 12 | 7.30E-05 | 1.44 |
| *SLC25A19* | solute carrier family 25 , member 19 | 2.90E-05 | 1.44 |
| *HAX1* | HCLS1 associated protein X-1 | 4.30E-05 | 1.44 |
| *TRAP1* | TNF receptor-associated protein 1 | 5.62E-05 | 1.44 |
| *WDR75* | WD repeat domain 75 | 2.81E-05 | 1.43 |
| *ADAT2* | adenosine deaminase, tRNA-specific 2, TAD2 homolog | 2.90E-05 | 1.43 |
| *AP1S1* | adaptor-related protein complex 1, sigma 1 subunit | 2.81E-05 | 1.43 |
| *MAPRE1* | microtubule-associated protein, RP/EB family, member 1 | 2.90E-05 | 1.43 |
| *MSRB1* | methionine sulfoxide reductase B1 | 2.81E-05 | 1.42 |
| *ALG3* | asparagine-linked glycosylation 3,) | 3.68E-05 | 1.42 |
| *PIGM* | phosphatidylinositol glycan anchor biosynthesis, class M | 1.53E-05 | 1.42 |
| *MDN1* | MDN1, midasin homolog | 2.81E-05 | 1.42 |
| *NANP* | N-acetylneuraminic acid phosphatase | 2.81E-05 | 1.41 |
| *SUCO* | SUN domain containing ossification factor | 2.81E-05 | 1.41 |
| *FBXO28* | F-box protein 28 | 2.81E-05 | 1.41 |
| *SNX11* | sorting nexin 11 | 6.96E-05 | 1.41 |
| *FDFT1* | farnesyl-diphosphate farnesyltransferase 1 | 7.29E-05 | 1.41 |
| *SUPT4H1* | suppressor of Ty 4 homolog 1 | 2.81E-05 | 1.41 |
| *MTMR3* | myotubularin related protein 3 | 2.90E-05 | 1.40 |
| *PPME1* | protein phosphatase methylesterase 1 | 2.90E-05 | 1.40 |
| *DPAGT1* | dolichyl-phosphate -N-acetylglucosaminephosphotransferase 1 | 2.90E-05 | 1.40 |
| *NUP85* | nuclear undecaprenyl pyrophosphate synthase 1 | 5.60E-05 | 1.40 |
| *RRAGA* | Ras-related GTP binding A | 1.07E-05 | 1.40 |
| *BLMH* | bleomycin hydrolase | 2.81E-05 | 1.40 |
| *AP4S1* | adaptor-related protein complex 4, sigma 1 subunit | 9.43E-05 | 1.39 |
| *NOP58* | NOL1/NOP2/Sun domain family, member 2 | 2.81E-05 | 1.39 |
| *GNE* | glucosamine (UDP-N-acetyl)-2-epimerase/N-acetylmannosamine kinase | 2.81E-05 | 1.39 |
| *CAND1* | cullin-associated and neddylation-dissociated 1 | 1.53E-05 | 1.39 |
| *ATAD1* | ATPase family, AAA domain containing 1 | 7.49E-05 | 1.39 |
| *FAM20B* | family with sequence similarity 20, member B | 2.81E-05 | 1.39 |
| *EI24* | etoposide induced 2.4 mRNA | 2.90E-05 | 1.39 |
| *SLC39A9* | solute carrier family 39 (zinc transporter), member 9 | 2.81E-05 | 1.39 |
| *STARD7* | StAR-related lipid transfer (START) domain containing 7 | 2.90E-05 | 1.39 |
| *HYOU1* | hypoxia up-regulated 1 | 5.04E-05 | 1.39 |
| *WAPAL* | wings apart-like homolog | 2.81E-05 | 1.38 |
| *MINPP1* | multiple inositol polyphosphate histidine phosphatase, 1 | 2.04E-05 | 1.38 |
| *NOP16* | NOP58 ribonucleoprotein homolog | 5.50E-05 | 1.38 |
| *USP8* | ubiquitin specific peptidase 8 | 2.81E-05 | 1.37 |
| *CTR9* | Ctr9, Paf1/RNA polymerase II complex component, homolog (Scerevisiae) | 3.32E-05 | 1.37 |
| *IBTK* | inhibitor of Bruton agammaglobulinemia tyrosine kinase | 5.62E-05 | 1.37 |
| *FAM136A* | family with sequence similarity 136, member A | 2.90E-05 | 1.37 |
| *DNAJC16* | DnaJ (Hsp40) homolog, subfamily C, member 16 | 2.81E-05 | 1.37 |
| *COPB2* | coatomer protein complex, subunit beta 2 (beta prime) | 2.90E-05 | 1.37 |
| *SUMF2* | sulfatase modifying factor 2 | 3.85E-05 | 1.36 |
| *NSUN2* | nudix (nucleoside diphosphate linked moiety X)-type motif 9 | 2.90E-05 | 1.36 |
| *EPRS* | glutamyl-prolyl-tRNA synthetase | 2.90E-05 | 1.36 |
| *ENTPD6* | ectonucleoside triphosphate diphosphohydrolase 6 | 5.62E-05 | 1.35 |
| *TSFM* | Ts translation elongation factor, mitochondrial | 3.04E-05 | 1.35 |
| ***Continued*** |  |  |  |
| ***Gene*** | **Gene Name** | **qValue** | **FC** |
| *AHSA1* | AHA1, activator of heat shock 90kDa protein ATPase homolog 1 (yeast) | 5.94E-05 | 1.35 |
| *KPTN* | kaptin (actin binding protein) | 7.49E-05 | 1.35 |
| *ACTR2* | ARP2 actin-related protein 2 homolog | 5.62E-05 | 1.35 |
| *DDX18* | DEAD (Asp-Glu-Ala-Asp) box polypeptide 18 | 2.81E-05 | 1.35 |
| *CMTM6* | CKLF-like MARVEL transmembrane domain containing 6 | 3.16E-05 | 1.35 |
| *IARS* | isoleucyl-tRNA synthetase | 3.04E-05 | 1.35 |
| *USP10* | ubiquitin specific peptidase 10 | 2.90E-05 | 1.34 |
| *DNAJC5* | DnaJ (Hsp40) homolog, subfamily C, member 5 | 2.81E-05 | 1.34 |
| *RNF121* | ring finger protein 121 | 2.81E-05 | 1.34 |
| *FLVCR2* | feline leukemia virus subgroup C cellular receptor family, member 2 | 2.81E-05 | 1.33 |
| *NUP43* | nucleoporin 85kDa | 2.81E-05 | 1.33 |
| *FAM63B* | family with sequence similarity 63, member B | 2.81E-05 | 1.33 |
| *MYL12A* | myosin, light chain 12A, regulatory, non-sarcomeric | 8.13E-05 | 1.33 |
| *AK2* | adenylate kinase 2 | 5.04E-05 | 1.33 |
| *SF3B3* | splicing factor 3b, subunit 3, 130kDa | 4.45E-05 | 1.32 |
| *TTC37* | tetratricopeptide repeat domain 37 | 1.07E-05 | 1.32 |
| *WDR92* | WD repeat domain 92 | 3.32E-05 | 1.32 |
| *SLC35A4* | solute carrier family 35, member A4 | 2.01E-05 | 1.32 |
| *RAB22A* | RAB22A, member RAS oncogene family | 2.47E-05 | 1.32 |
| *HMGN2* | hypothetical LOC729505; similar to hCG2040565 | 5.70E-05 | 1.32 |
| *HMGXB3* | HMG box domain containing 3 | 3.20E-05 | 1.32 |
| *AKAP1* | A kinase (PRKA) anchor protein 1 | 7.73E-05 | 1.31 |
| *RAB2B* | RAB2B, member RAS oncogene family | 2.90E-05 | 1.31 |
| *SEPHS1* | selenophosphate synthetase 1; similar to selenophosphate synthetase 1 | 2.81E-05 | 1.31 |
| *WDR55* | WD repeat domain 55 | 2.01E-05 | 1.31 |
| *VPS4A* | vacuolar protein sorting 4 homolog A | 4.08E-05 | 1.31 |
| *ZNF259* | zinc finger protein 259 | 4.87E-05 | 1.31 |
| *CLDN12* | claudin 12 | 2.81E-05 | 1.30 |
| *G3BP1* | GTPase activating protein (SH3 domain) binding protein 1 | 5.56E-05 | 1.30 |
| *LRRC42* | leucine rich repeat containing 42 | 2.81E-05 | 1.30 |
| *GALNT11* | UDP-N-acetyl-alpha-D-galactosamine:polypeptide N-acetylgalactosaminyltransferase 11 | 2.81E-05 | 1.30 |
| *ARPC5* | actin related protein 2/3 complex, subunit 5, 16kDa | 2.04E-05 | 1.29 |
| *AVEN* | apoptosis, caspase activation inhibitor | 5.04E-05 | 1.28 |
| *THADA* | thyroid adenoma associated | 6.76E-05 | 1.27 |
| *FAS* | Fas (TNF receptor superfamily, member 6) | 4.70E-05 | 1.25 |
| *YWHAG* | tyrosine 3-monooxygenase/tryptophan 5-monooxygenase activation protein, gamma polypeptide | 2.90E-05 | 1.25 |
| *C7orf25* | chromosome 7 open reading frame 25 | 2.90E-05 | 1.25 |
| *SNX4* | sorting nexin 4 | 3.16E-05 | 1.25 |
| *RAB1A* | RAB1A, member RAS oncogene family | 2.90E-05 | 1.24 |
| *ISCA2* | iron-sulfur cluster assembly 2 homolog | 2.90E-05 | 1.24 |
| *PTAR1* | protein prenyltransferase alpha subunit repeat containing 1 | 5.11E-05 | 1.23 |
| *MTMR9* | myotubularin related protein 9 | 7.49E-05 | 1.23 |
| *PIKFYVE* | phosphoinositide kinase, FYVE finger containing | 5.04E-05 | 1.22 |
| *PGAP3* | post-GPI attachment to proteins 3 | 5.04E-05 | 1.20 |
| *CDC42SE2* | CDC42 small effector 2 | 2.81E-05 | 1.19 |
| *KIAA1274* | KIAA1274 | 2.81E-05 | 1.15 |
| *LOC100126544* | uncaracterirazed | 3.04E-05 | 1.04 |
