# Supplementary figures and images for: Genome-wide expression profiling in muscle and subcutaneous fat of lambs in response to the intake of concentrate supplemented with vitamin E
Source: BMC Genomics. 2017 Jan 17;18:92. doi: 10.1186/s12864-016-3405-8 (PMC5240399; doi:10.1186/s12864-016-3405-8)

**Supplementary Fig. S1**. Hierarchical clustering analysis in subcutaneous fat using 330 SAM genes.


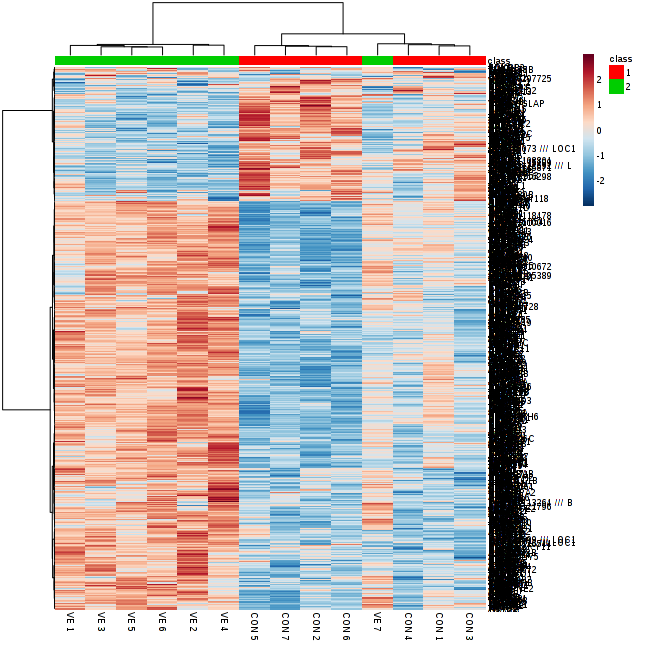

Supplement: Additional file 2: Figure S1. — Hierarchical clustering analysis in subcutaneous fat using 330 SAM genes. (DOCX 290 kb) [file 12864_2016_3405_MOESM2_ESM.docx]
