# Supplementary material for: Genome-wide expression profiling in muscle and subcutaneous fat of lambs in response to the intake of concentrate supplemented with vitamin E
Source: BMC Genomics. 2017 Jan 17;18:92. doi: 10.1186/s12864-016-3405-8 (PMC5240399; doi:10.1186/s12864-016-3405-8)
Supplement: Additional file 3: Table S2. — DAVID Functional Annotation Clustering of SAM genes in VE vs. CON muscle L. Thoracis. Only is shown the 2 most enrichment cluster. (DOCX 16 kb) [file 12864_2016_3405_MOESM3_ESM.docx]

| **Annotation Cluster 1** | **Enrichment Score: 0.6128986664420012** | | | |  | |  |  |
| --- | --- | --- | --- | --- | --- | --- | --- | --- |
| **Category** | **Term** | **Count** | **%** | **PValue** | | **Genes** | |  |
| GOTERM_BP_FAT | GO:0007242~intracellular signaling cascade | 4 | 19.05 | 0.13 | | IGF1R, DEF8, AKAP7, CISH | |  |
| GOTERM_BP_ALL | GO:0007242~intracellular signaling cascade | 4 | 19.05 | 0.17 | | IGF1R, DEF8, AKAP7, CISH | |  |
| GOTERM_BP_ALL | GO:0007165~signal transduction | 4 | 19.05 | 0.65 | | IGF1R, DEF8, AKAP7, CISH | |  |
|  |  |  |  |  | |  | |  |
| **Annotation Cluster 2** | **Enrichment Score: 0.6020566970277776** | | |  | |  | |  |
| GOTERM_BP_ALL | GO:0008152~metabolic process | 14 | 66.67 | 0.02 | | SAT1, ZNF79, MAFB, MYLK2, ACACB, ACAT1, CISH, IGF1R, PGLS, DUSP26, AKR7A2, FBXL4, AKAP7, RSC1A1 | |  |
| GOTERM_BP_ALL | GO:0044237~cellular metabolic process | 12 | 57.14 | 0.07 | | IGF1R, PGLS, ZNF79, DUSP26, MAFB, AKR7A2, MYLK2, FBXL4, AKAP7, ACACB, CISH, RSC1A1 | |  |
| GOTERM_BP_ALL | GO:0044238~primary metabolic process | 12 | 57.14 | 0.09 | | IGF1R, PGLS, ZNF79, DUSP26, MAFB, AKR7A2, MYLK2, FBXL4, AKAP7, ACACB, CISH, RSC1A1 | |  |
| GOTERM_BP_ALL | GO:0044260~cellular macromolecule metabolic process | 9 | 42.86 | 0.20 | | IGF1R, ZNF79, DUSP26, MAFB, MYLK2, FBXL4, AKAP7, CISH, RSC1A1 | |  |
| GOTERM_BP_ALL | GO:0043170~macromolecule metabolic process | 9 | 42.86 | 0.30 | | IGF1R, ZNF79, DUSP26, MAFB, MYLK2, FBXL4, AKAP7, CISH, RSC1A1 | |  |
| GOTERM_BP_ALL | GO:0031323~regulation of cellular metabolic process | 6 | 28.57 | 0.35 | | IGF1R, ZNF79, MAFB, ACACB, CISH, RSC1A1 | |  |
| GOTERM_BP_ALL | GO:0009987~cellular process | 14 | 66.67 | 0.39 | | H1F0, ZNF79, MAFB, MYLK2, ACACB, CISH, IGF1R, PGLS, DUSP26, AKR7A2, ABCC4, FBXL4, AKAP7, RSC1A1 | |  |
| GOTERM_BP_ALL | GO:0019222~regulation of metabolic process | 6 | 28.57 | 0.39 | | IGF1R, ZNF79, MAFB, ACACB, CISH, RSC1A1 | |  |
| GOTERM_BP_ALL | GO:0080090~regulation of primary metabolic process | 5 | 23.81 | 0.53 | | IGF1R, ZNF79, MAFB, ACACB, RSC1A1 | |  |
| GOTERM_BP_ALL | GO:0050794~regulation of cellular process | 9 | 42.86 | 0.54 | | IGF1R, ZNF79, MAFB, DEF8, MYLK2, AKAP7, ACACB, CISH, RSC1A1 | |  |
| GOTERM_BP_ALL | GO:0050789~regulation of biological process | 9 | 42.86 | 0.61 | | IGF1R, ZNF79, MAFB, DEF8, MYLK2, AKAP7, ACACB, CISH, RSC1A1 | |  |
| GOTERM_BP_ALL | GO:0065007~biological regulation | 9 | 42.86 | 0.69 | | IGF1R, ZNF79, MAFB, DEF8, MYLK2, AKAP7, ACACB, CISH, RSC1A1 | |  |

**Supplementary Table S2**. DAVID Functional Annotation Clustering of SAM genes in VE vs. CON muscle L. Thoracis. Only is shown the 2 most enrichment cluster.
